# Supplementary material for: Comorbidity and outcomes in traumatic brain injury: protocol for a systematic review on functional status and risk of death
Source: BMJ Open. 2017 Oct 13;7(10):e018626. doi: 10.1136/bmjopen-2017-018626 (PMC5652528; doi:10.1136/bmjopen-2017-018626)
Supplement: Supplementary file 1 [file bmjopen-2017-018626supp001.pdf]

Supplementary Table S1. Search details.
Searches conducted in Medline (including Medline in Process and other non-indexed citations, ePubs and Medline Daily), Embase, Cochrane Central Register of Controlled Trials, and PsycINFO. Searches were limited from 1997 to May 2017. Searches were limited to English language papers, and to an adult, human population when possible. Searches were conducted by an Information Specialist (JB).

PRE-DUPLICATE REMOVAL

TOTAL Results: 9414
Medline: 2860
Central: 178
Embase: 5468
PsycINFO: 908

POST-DUPLICATE REMOVAL

TOTAL Results: 7443
Duplicates removed: 1971
Medline: 2748
Central: 116
Embase: 4088
PsycINFO: 491

Database: Ovid MEDLINE(R) Epub Ahead of Print, In-Process & Other Non-Indexed Citations, Ovid MEDLINE(R) Daily and Ovid MEDLINE(R) <1946 to Present>
Search Strategy:
----
1 exp brain injuries/ (59169)
2 Craniocerebral Trauma/ (21199)
3 exp Head Injuries, Closed/ (9232)
4 exp Skull Fractures/ (20700)
5 mTBI*2.tw,kw. (1835)
6 tbi*2.tw,kw. (20636)
7 concuss*.tw,kw. (6496)
8 ((head* or cerebr* or crani* or skull* or intracran*) adj2 (injur* or trauma* or damag* or wound* or swell* or oedema* or edema* or fracture* or contusion* or pressur*)).tw,kw. (75791)
9 ((brain* or cerebr* or intracerebr* or crani* or intracran* or head* or subdural* or epidural* or extradural*) adj (haematoma* or hematoma* or hemorrhag* or haemorrhag* or bleed*)).tw,kw. (42638)
10 or/1-9 (185742)
11 exp Comorbidity/ (88810)
12 exp Risk Adjustment/ (2770)
13 (comorbid* or co morbid* or multimorbid* or multi morbid*).tw,kw. (131913)
14 (polypatholog* or poly-patholog*).tw,kw. (151)
15 ((clinical* or medical*) adj3 complex*).tw,kw. (11314)

16 ((coexist* or co exist* or cooccur* or co-occur* or multipl*) adj3 (illness* or disease* or disorder* or condition* or complication* or diagnos* or risk*)).tw,kw. (69114)
17 (multidisease? or multi-disease? or (multiple adj (ill* or disease? or condition? or syndrom* or disorder?))).tw,kw. (3721)
18 ((several* or various or (two adj2 more) or concomitant or conjoined or concurrent) adj3 (morbid* or ill* or disease* or sick* or condition*)).tw,kw. (129147)
19 "comorbidity-polypharmacy score".tw,kw. (11)
20 ('charlson comorbidity index' or 'CCI' or 'CMI' or elixhauser or 'BOD index' or 'cumulative index rating scale' or 'CIRS' or 'Coroni-Huntley index' or 'DUSOI index' or 'Hallstrom index' or 'Hurwitz index' or 'Incalzi index', 'Kaplan index', 'Liu index', 'Shwartz index' or 'comorbidity-polypharmacy score').tw,kw. (10394)
21 or/11-20 (394772)
22 10 and 21 (5122)
23 exp Mortality/ (335478)
24 exp morbidity/ (467773)
25 (morbidit* or mortalit*).tw,kw. (760891)
26 function*.mp. (3282516)
27 or/23-26 (4495784)
28 exp cohort studies/ (1680971)
29 exp prognosis/ (1379668)
30 exp survival analysis/ (241795)
31 exp models, statistical/ (344993)
32 prognos*.tw,kw. (492733)
33 predict*.tw,kw. (1261259)
34 course*.tw,kw. (546731)
35 diagnosed.tw,kw. (461120)
36 cohort*.tw,kw. (423751)
37 death.tw,kw. (584394)
38 exp treatment outcome/ (841041)
39 "early termination of clinical trials"/ (544)
40 treatment failure/ (31446)
41 incidence/ (222806)
42 or/28-41 (5055925)
43 27 or 42 (8021818)
44 22 and 43 (3756)
45 44 not (exp animals/ not exp humans/) (3301)
46 45 not (exp children/ not exp adults/) (3118)
47 limit 46 to english language (2860)
48 remove duplicates from 47 (2750)

****

Database: Embase <1974 to 2017 May 10>
Search Strategy:
----
1 exp brain injury/ (148571)

2 head injury/ (44023)
3 mTBI*2.tw. (2763)
4 tbi*2.tw. (32342)
5 concuss*.tw. (7543)
6 ((head* or cerebr* or crani* or skull* or intracran*) adj2 (injur* or trauma* or damag* or wound* or swell* or oedema* or edema* or fracture* or contusion* or pressur*)).tw,kw. (96428)
7 ((brain* or cerebr* or intracerebr* or crani* or intracran* or head* or subdural* or epidural* or extradural*) adj (haematoma* or hematoma* or hemorrhag* or haemorrhag* or bleed*)).tw,kw. (59251)
8 or/1-7 (291625)
9 comorbidity/ (177225)
10 risk assessment/ (409214)
11 exp comorbidity assessment/ (9540)
12 (comorbid* or co morbid* or multimorbid* or multi morbid*).tw,kw. (217271)
13 (polypatholog* or poly-patholog*).tw,kw. (290)
14 ((clinical* or medical*) adj3 complex*).tw,kw. (15500)
15 ((coexist* or co exist* or cooccur* or co-occur* or multipl*) adj3 (illness* or disease* or disorder* or condition* or complication* or diagnos* or risk*)).tw,kw. (94039)
16 (multidisease? or multi-disease? or (multiple adj (ill* or disease? or condition? or syndrom* or disorder?))).tw,kw. (4710)
17 ((several* or various or (two adj2 more) or concomitant or conjoined or concurrent) adj3 (morbid* or ill* or disease* or sick* or condition*)).tw,kw. (163497)
18 "comorbidity-polypharmacy score".tw,kw. (15)
19 ('charlson comorbidity index' or 'CCI' or 'CMI' or elixhauser or 'BOD index' or 'cumulative index rating scale' or 'CIRS' or 'Coroni-Huntley index' or 'DUSOI index' or 'Hallstrom index' or 'Hurwitz index' or 'Incalzi index', 'Kaplan index', 'Liu index', 'Shwartz index' or 'comorbidity-polypharmacy score').tw,kw. (19018)
20 or/9-19 (943533)
21 8 and 20 (15388)
22 exp mortality/ (858109)
23 exp morbidity/ (299827)
24 (morbidit* or mortalit*).tw,kw. (1038507)
25 function*.mp. (4279406)
26 or/22-25 (5472712)
27 cohort analysis/ (287239)
28 prognosis/ (506894)
29 survival analysis/ (3712)
30 exp survival/ (845725)
31 statistical model/ (136685)
32 exp "prediction and forecasting"/ (1010760)
33 prognos*.tw,kw. (700803)
34 predict*.tw,kw. (1580756)
35 course*.tw,kw. (702204)
36 diagnosed.tw,kw. (688998)

37 cohort*.tw,kw. (649460)
38 death.tw,kw. (778611)
39 exp treatment outcome/ (1236966)
40 "early termination of clinical trial"/ (204)
41 exp treatment failure/ (108708)
42 exp incidence/ (330423)
43 or/27-42 (5901375)
44 26 or 43 (9681606)
45 21 and 44 (10931)
46 45 not ((exp animals/ or exp animal experimentation/ or nonhuman/) not exp human/) (9877)
47 46 not ((exp embryo/ or exp fetus/ or exp juvenile/) not exp adult/) (8968)
48 limit 47 to english language (8463)
49 limit 48 to embase (5871)
50 limit 49 to (conference abstract or conference paper or "conference review") (179)
51 49 not 50 (5692)
52 limit 51 to yr="1997 -Current" (5468)

****

Database: EBM Reviews - Cochrane Central Register of Controlled Trials <April 2017>
Search Strategy:
----
1 exp brain injuries/ (1135)
2 Craniocerebral Trauma/ (247)
3 exp Head Injuries, Closed/ (167)
4 exp Skull Fractures/ (192)
5 mTBI*2.tw,kw. (122)
6 tbi*2.tw,kw. (1306)
7 concuss*.tw,kw. (221)
8 ((head* or cerebr* or crani* or skull* or intracran*) adj2 (injur* or trauma* or damag* or wound* or swell* or oedema* or edema* or fracture* or contusion* or pressur*)).tw,kw. (3278)
9 ((brain* or cerebr* or intracerebr* or crani* or intracran* or head* or subdural* or epidural* or extradural*) adj (haematoma* or hematoma* or hemorrhag* or haemorrhag* or bleed*)).tw,kw. (3605)
10 or/1-9 (8539)
11 exp Comorbidity/ (3012)
12 exp Risk Adjustment/ (20)
13 (comorbid* or co morbid* or multimorbid* or multi morbid*).tw,kw. (9750)
14 (polypatholog* or poly-patholog*).tw,kw. (4)
15 ((clinical* or medical*) adj3 complex*).tw,kw. (711)
16 ((coexist* or co exist* or cooccur* or co-occur* or multipl*) adj3 (illness* or disease* or disorder* or condition* or complication* or diagnos* or risk*)).tw,kw. (4140)
17 (multidisease? or multi-disease? or (multiple adj (ill* or disease? or condition? or syndrom* or disorder?))).tw,kw. (107)

18 ((several* or various or (two adj2 more) or concomitant or conjoined or concurrent) adj3 (morbid* or ill* or disease* or sick* or condition*)).tw,kw. (1145)
19 "comorbidity-polypharmacy score".tw,kw. (0)
20 ('charlson comorbidity index' or 'CCI' or 'CMI' or elixhauser or 'BOD index' or 'cumulative index rating scale' or 'CIRS' or 'Coroni-Huntley index' or 'DUSOI index' or 'Hallstrom index' or 'Hurwitz index' or 'Incalzi index', 'Kaplan index', 'Liu index', 'Shwartz index' or 'comorbidity-polypharmacy score').tw,kw. (748)
21 or/11-20 (17592)
22 10 and 21 (214)
23 22 not (exp animals/ not exp humans/) (214)
24 23 not (exp Child/ not exp Adult/) (213)
25 limit 24 to english language (185)
26 limit 25 to yr="1997 -Current" (178)

****

Database: PsycINFO <1806 to May Week 1 2017>
Search Strategy:
----
1 exp traumatic brain injury/ (15677)
2 exp head injuries/ (5584)
3 mTBI*2.tw. (1312)
4 tbi*2.tw. (8651)
5 concuss*.tw. (2360)
6 ((head* or cerebr* or crani* or skull* or intracran*) adj2 (injur* or trauma* or damag* or wound* or swell* or oedema* or edema* or fracture* or contusion* or pressur*)).tw. (11318)
7 ((brain* or cerebr* or intracerebr* or crani* or intracran* or head* or subdural* or epidural* or extradural*) adj (haematoma* or hematoma* or hemorrhag* or haemorrhag* or bleed*)).tw. (3029)
8 or/1-7 (28382)
9 comorbidity/ (27127)
10 risk assessment/ (11895)
11 (comorbid* or co morbid* or multimorbid* or multi morbid*).tw. (49497)
12 (polypatholog* or poly-patholog*).tw. (23)
13 ((clinical* or medical*) adj3 complex*).tw. (2577)
14 ((coexist* or co exist* or cooccur* or co-occur* or multipl*) adj3 (illness* or disease* or disorder* or condition* or complication* or diagnos* or risk*)).tw. (16826)
15 (multidisease? or multi-disease? or (multiple adj (ill* or disease? or condition? or syndrom* or disorder?))).tw. (666)
16 ((several* or various or (two adj2 more) or concomitant or conjoined or concurrent) adj3 (morbid* or ill* or disease* or sick* or condition*)).tw. (14708)
17 "comorbidity-polypharmacy score".tw. (2)
18 ('charlson comorbidity index' or 'CCI' or 'CMI' or elixhauser or 'BOD index' or 'cumulative index rating scale' or 'CIRS' or 'Coroni-Huntley index' or 'DUSOI index' or

'Hallstrom index' or 'Hurwitz index' or 'Incalzi index', 'Kaplan index', 'Liu index', 'Shwartz index' or 'comorbidity-polypharmacy score').tw,tm. (2701)
19 or/9-18 (97253)
20 8 and 19 (1354)
21 limit 20 to animal (317)
22 limit 20 to human (1042)
23 20 not (21 not 22) (1071)
24 limit 23 to ((childhood <birth to 12 years> or adolescence <13 to 17 years>) and (100 childhood <birth to age 12 yrs> or 120 neonatal <birth to age 1 mo> or 140 infancy <2 to 23 mo> or 160 preschool age <age 2 to 5 yrs> or 180 school age <age 6 to 12 yrs> or 200 adolescence <age 13 to 17 yrs>)) (132)
25 limit 23 to (adulthood <18+ years> and ("300 adulthood <age 18 yrs and older>" or 320 young adulthood <age 18 to 29 yrs> or 340 thirties <age 30 to 39 yrs> or 360 middle age <age 40 to 64 yrs> or "380 aged <age 65 yrs and older>" or "390 very old <age 85 yrs and older>")) (589)
26 23 not (24 not 25) (1023)
27 limit 26 to english language (977)
28 limit 27 to yr="1997 -Current" (908)

****
