# Supplementary material for: Comorbidity and outcomes in traumatic brain injury: protocol for a systematic review on functional status and risk of death
Source: BMJ Open. 2017 Oct 13;7(10):e018626. doi: 10.1136/bmjopen-2017-018626 (PMC5652528; doi:10.1136/bmjopen-2017-018626)
Supplement: Author's manuscript [file bmjopen-2017-018626.draft_revisions.pdf]

BMJ Open is committed to open peer review. As part of this commitment we make the peer review history of every article we publish publicly available.

When an article is published we post the peer reviewers' comments and the authors' responses online. We also post the versions of the paper that were used during peer review. These are the versions that the peer review comments apply to.

The versions of the paper that follow are the versions that were submitted during the peer review process. They are not the versions of record or the final published versions. They should not be cited or distributed as the published version of this manuscript.

BMJ Open is an open access journal and the full, final, typeset and author-corrected version of record of the manuscript is available on our site with no access controls, subscription charges or pay-per-view fees (http://bmjopen.bmj.com).

If you have any questions on BMJ Open's open peer review process please email editorial.bmjopen@bmj.com

BMJ Open

Comorbidity and outcomes in traumatic brain injury: Protocol for a systematic review on functional status and risk of death.

Journal:	*BMJ Open*
Manuscript ID	bmjopen-2017-018626
Article Type:	Protocol
Date Submitted by the Author:	10-Jul-2017
Complete List of Authors:	Mollayeva , Tatyana; Toronto Rehabilitation Institute, Research; University of Toronto, Rehabilitation Sciences Institute<br>Xiong, Chen; Rehabilitation Sciences Institute , University of Toronto<br>Hanafy, Sara; Toronto Rehabilitation Institute-University Health Network, Research<br>Chan , Vincy ; Toronto Rehabilitation Institute , Research<br>Hu, Zheng Jing ; Toronto Rehabilitation Institute-University Health Network, Research<br>Sutton, Mitchell; Toronto Rehabilitation Institute-University Health Network, Research<br>Escobar, Michael ; University of Toronto Dalla Lana School of Public Health<br>Colantonio, Angela; University of Toronto, Rehabilitation Science Institute
**Primary Subject Heading**:	Epidemiology
Secondary Subject Heading:	Research methods, Neurology
Keywords:	comorbidity, traumatic brain injury, prognostic review, mortality, cognitive/physical status, sex and age stratification

SCHOLARONE™ Manuscripts

For peer review only - http://bmjopen.bmj.com/site/about/guidelines.xhtml

TITLE: Comorbidity and outcomes in traumatic brain injury: Protocol for a systematic review on functional status and risk of death.

Authors: Tatyana Mollayeva, MD, PhD [1-3], Chen Xiong, MSc[1-3], Sara Hanafy, MSc[2,3], Vincy Chan, MPH, PhD[2-4], Zheng Jing Hu, MSc[5], Mitchell Sutton, MSc[5], Michael Escobar, PhD[5], Angela Colantonio, PhD [1-3,5]

Author details

[1]Rehabilitation Sciences Institute, Faculty of Medicine, University of Toronto, Canada

[2]Toronto Rehabilitation Institute-University Health Network, Canada

[3]Aquired Brain Injury Research Lab, University of Toronto, Canada

[4] Pediatric Oncology Group of Ontario, Canada

[5]Dalla Lana School of Public Health, University of Toronto

Tatyana Mollayeva, MD, PhD (corresponding author)

Rehabilitation Sciences Institute

University of Toronto

Toronto Rehabilitation Institute-University Health Network

550 University Avenue, Rm 11207

Toronto, Ontario M5G 2A2

tatyana.mollayeva@utoronto.ca

tel: 416-597-3422 ext 7848

fax: 416-946-8570

Chen Xiong, MSc, PhD student

For peer review only

Rehabilitation Sciences Institute

Rehabilitation Sciences Building, University of Toronto

500 University Avenue, Room 260

Toronto, ON M5G 1V7

chen.xiong@mail.utoronto.ca

tel: 647-770-8366

fax: 416-946-8570

Sara Hanafy, MSc

Researcher-volunteer

Toronto Rehabilitation Institute-University Health Network

550 University Avenue

Toronto, ON M5G 2A2

sara.hanafy@utoronto.ca

tel: 416-597-3422

fax: 416-946-8570

Vincy Chan, PhD, MPH

Pediatric Oncology Group of Ontario, Canada

Toronto Rehabilitation Institute-University Health Network

550 University Avenue

Toronto, ON, M5G 2A2

vincy.chan@uhn.ca

For peer review only

416-597-3422 ext. 7845

Zheng Jing Hu, MSc

Summary research intern

Toronto Rehabilitation Institute-University Health Network

550 University Avenue, Rm 11-207

Toronto, Ontario M5G 2A2

Dalla Lana School of Public Health

University of Toronto

Zhengjing.hu@mail.utoronto.ca

tel: 416-597-3422

fax: 416-946-8570

Mitchell Sutton, MSc

Summer research intern

Toronto Rehabilitation Institute-University Health Network

550 University Avenue, Rm 11-207

Toronto, Ontario M5G 2A2

Dalla Lana School of Public Health

University of Toronto

mitchell.sutton@mail.utoronto.ca

tel: 416-597-3422

fax: 416-946-8570

For peer review only

Michael Escobar, PhD

Professor, Dalla Lana School of Public Health

Health Sciences Building

155 College Street, 6th Floor

Toronto, ON M5T 3M7

m.escobar@utoronto.ca

tel: 416- 978-7942

fax: 416-946-8570

Angela Colantonio, PhD

Director, Rehabilitation Sciences Institute

University of Toronto

Senior Scientist, Toronto Rehabilitation Institute-University Health Network

160-500 University Ave.

Toronto, Ontario M5G 1V7

angela.colantonio@utoronto.ca

tel: 416-978-1098

fax: 416-946-8570

For peer review only

ABSTRACT

Introduction: Reports on the association between comorbidity and functional status and risk of death in patients with traumatic brain injury (TBI) have been inconsistent; it is currently unknown which additional clinical entities (comorbidities) have an adverse influence on the evolution of outcomes across the lifespan of males and females with TBI. The current protocol outlines a strategy for a systematic review of the current evidence examining the impact of comorbidity on functional status and early and late-term mortality, taking into account known risk factors of these adverse outcomes (i.e., demographic (age and sex), and injury-related characteristics).

Methods and analysis: A comprehensive search strategy for TBI prognosis, functional (cognitive and physical) status and mortality studies has been developed in collaboration with a medical information specialist of the large rehabilitation teaching hospital. All peer-reviewed English language studies with longitudinal design in adults with TBI of any severity, published from May 1997 to April 2017, found through Medline, Central, Embase, Scopus, PsycINFO and bibliographies of identified articles, will be considered eligible. Study quality will be assessed using published guidelines.

Ethics and dissemination: The number of males and females of all ages surviving TBI progressively increases. While the neurological consequences of TBI are well described, evidence is emerging on associations between comorbid disorders and adverse short and long-term outcomes post-injury. The significant economic and human costs of TBI merit the call for systematic efforts to understand all factors that contribute to these adverse outcomes to inform risk stratification of patients to guide the management of brain injury acutely and at the chronic stages post-injury on a population level. *Registration details:* CRD42017070033.

For peer review only

Keywords: comorbidity; traumatic brain injury; prognostic review; mortality; cognitive status; physical status; age; sex

For peer review only

Strengths and limitations of this study

- The study of comorbidity in traumatic brain injury (TBI) is important as any additional clinical entity can change future life course and injury outcomes
- To date, there has been no systematic review on the topic of comorbidity in TBI as it relates to all-cause mortality and functional status post injury; the protocol outlines a strategy for a study that intends to fill the gap
- Attention to known risk factors of adverse outcomes such as sex, age, and TBI severity will permit advanced risk stratification and inform future prognostic studies
- Biases associated with unequal sex and age distribution, residual confounding due to TBI- or comorbidity-related treatment effect could not be avoided
- Systematizing prognostic data on comorbidity in TBI is essential for patients, health care providers, policymakers, and health researchers.

For peer review only

Introduction

Traumatic Brain Injury (TBI), defined as "*an alteration in brain function, or other evidence of brain pathology, caused by an external force,"* [1]is a major global health concern. According to the World Health Organization, death and disability from TBI is rising rapidly.[2] In the US, the total annual cost of TBI was estimated to be $60.43 billion US dollars.[3] In Canada, a recent report by the Public Health Agency projected that the indirect economic cost of a TBI due to working-age death and disability will increase from $7.3 billion in 2011 to $8.2 billion by 2031, far exceeding that of other common neurological conditions (e.g., epilepsy, multiple sclerosis, Alzheimer's Disease, combined, with estimated $4.8 billion in 2011 and $5.8 billion in 2031, respectively).[4] TBI may also exacerbate pre-existing disorders, or expedite the development of, additional clinical conditions in both the older and younger populations, increasing direct and indirect costs associated with TBI.[5]

Of particular importance is that the presence of a comorbidity (i.e., additional disease or illness coexisting with an index disease [6]) or multiple comorbidities in patients with TBI is common, [7-9] and is associated with high rates of hospitalizations, decreased functional status, and all-cause mortality.[7-9] Studies have also shown that comorbid disorders may alter the treatment course of patients with TBI by affecting the treatments that these patients receive in both the acute and rehabilitation setting.[7-9] Specifically, among patients that present with a comorbid health condition, treating or managing comorbidities are often prioritized over addressing TBI itself .[10-12] Likewise, the presence of any chronic comorbidity in TBI may lead patients to consume a disproportionate amount of healthcare resources .[13-14] While previous research has documented number of comorbidities in patients with TBI,[15-17] it is currently unknown which comorbid disorders pre-exist in TBI across ages, develop over time, and which best predict outcomes

For peer review only

related to functional (cognitive and physical) status and early and late post-injury mortality. It is also unknown if the presence of comorbidity changes the effect of traditional TBI risk factors for these outcomes, such as TBI severity and mechanism.[18-21]

Finally, it must be highlighted that a patient's sex and age has also been shown to drive differences in early mortality and functional recovery.[22-23] Previous research highlighted that case fatality ratios are elevated in older patients (60+ years of age) compared to younger patients, with sex differences observed in individuals 20 to 39 years of age .[23] Females are also at greater risk for the development of somatic and psychiatric comorbidity and associated functional decline post injury .[24] Overall, despite the strong evidence that comorbidities lead to adverse outcomes and complications among patients with TBI, a data synthesis on this topic taking sex and age into account does not exist to date. This highlights the need to explore how age and sex associate with comorbidity risk factors in patients with TBI.

As such, this protocol is for a systematic review on the topic of comorbidity in adult patients with TBI that aims to: (1) examine the relationship between comorbid disorder(s) and change in function after TBI and death; (2) determine the prognostic value of clinical characteristics of patients with TBI at baseline on the development of short, intermediate and long term adverse or beneficial outcome (s); and (3) review effects of comorbidity in the light of currently known risk factors of adverse outcomes (i.e., sex, age, and injury severity).

Methods and analysis

The systematic review that this protocol describes will be conducted and reported in compliance with the Preferred Reporting Items for Systematic Reviews and Meta-Analyses (PRISMA) guidelines.[25] In accordance with these guidelines, this systematic review protocol was registered

For peer review only - http://bmjopen.bmj.com/site/about/guidelines.xhtml

with the International Prospective Register of Systematic Reviews (PROSPERO) on June 22, 2017.[26]

Data sources and searches

In collaboration with TBI and rehabilitation experts, and a Medical Information Specialist, a comprehensive search strategy for prognostic studies of TBI outcomes (i.e., functional status and mortality) was developed (Table 1). All English language peer-reviewed studies published between March 1997 to April 2017, with prospective or retrospective data collection and a longitudinal design, found through Medline, Central, Embase, Scopus, PsycINFO and bibliographies of identified articles will be included. Reference lists of included studies will be reviewed to identify any additional relevant studies. Search terms for each database are presented in Table S1.

Comorbidity definition

Feinstein defined comorbidity as "the existence or occurrence of any distinct additional entity during the clinical course of a patient who has the index disease under study".[5] More recently, Valderas et al. defined it as "any additional condition that may occur during the clinical course of a patient who has an index condition that is the focus of interest".[27] Currently, there is no gold-standard for assessing comorbidity in TBI patients and reports of comorbidity vary widely in the published work .[18-21] In population based studies, comorbid conditions can be identified according to the International Classification (ICD) diagnostic codes; or converted into summary comorbidity measures focused on selected conditions, such as the Charlson Comorbidity Index, Aggregated Diagnosis Groups (ADG), Elixhauser Index, or the Total Illness Burden Index .[18-21] However, many papers focus on a single count of previously diagnosed chronic diseases that have shown a significant relation with death or functional outcomes in TBI population .[13-16]

For peer review only - http://bmjopen.bmj.com/site/about/guidelines.xhtml

Given that there is no consensus on the most appropriate method to construct comorbidity in TBI or whether one type is preferred to another, this systematic review will set restrictions only towards comorbid disorders being diagnosed, excluding self-report.

Predictors and outcomes

Predictors will be collated into three domains: sociodemographic characteristics, TBI-related characteristics, and comorbidity. All comorbidity-related variables (comorbidity index severity or presence of comorbidity) will be treated as primary predictors; hypothesized sociodemographic (age and sex) and TBI-related variables (injury severity, mechanism, and time since injury), if reported, will be considered as secondary predictors of our outcome(s) of interest. We will report on only those predictors that have been shown to be statistically significantly associated with our outcome(s) in at least one study and with reported quantitative data (i.e., event rates, risk ratios [RRs], odds ratios [ORs], or hazard ratios [HRs]) to measure the association between predictors and outcomes.

The following *outcomes* (either objectively documented or self-reported) will be considered: cognitive and physical status as assessed by the Glasgow Outcome Scale (GOS) with or without extended scores, Disability Rating Scale (DRS), Functional Independence Measure (FIM), the Functional Status Examination (FSE) or any other standardized functional measurement and mortality. In order for the previous objective (i.e., functional status) to be considered, the study has to define at least two time points' scores of functioning and/or has to provide details on when (at which time point since injury) a loss/gain/plato of function has been defined as a decline/improvement/stability. Should the definition of functional status vary amongst studies, the reported minimum change, expressed in percentage change, will be abstracted. For example, if the functional measure has a score in a range from zero to 10, and the minimum change

For peer review only - http://bmjopen.bmj.com/site/about/guidelines.xhtml

reported by researchers for the sample was one, we will assign a 10% ‘change’ on this group’s functional capabilities.

Inclusion and exclusion criteria

We will include studies that (1) primarily study comorbidity as it relates to our outcome(s) of interest; and (2) targeted adult patients (≥18 years of age) with a diagnosis of TBI on the basis of the accepted definitions (not self-report), and followed them for any period of time. Studies will neither be excluded based on the setting in which the research took place (acute care, rehabilitation setting, community, etc.) nor means of diagnosis of comorbidity. However, the following studies will be excluded: (1) more than 50% of participants had pre-existing TBIs or severe comorbidity (i.e., neurological or psychiatric diseases) at the baseline assessment, and if the subgroup with incident comorbidity and the patient outcome data could not be extracted independent of pre-existing cases (i.e., present before TBI); and (2) study designs/formats in letters to editors, reviews without data, case reports, or public reports, conference abstracts articles with no primary data, studies that focus on therapeutic interventions, and theses.

Zero-time (baseline assessment)

The nature of our objectives related to development of adverse outcomes in the TBI population (i.e., prognostic factors), raises the issue of zero-time bias. In prognostic studies, testing should start at a defined point, called zero time. Designated zero times (i.e. baseline or first assessment) vary between studies, [28] were majority of research from acute care/emergency studies performed baseline assessment within first month after the injury and majority of rehabilitation or community studies of prognosis performed baseline assessment prior to or at six-months post injury mark.[28] The limiter to zero-time has been set at six-month.

Study selection

Two independent researchers (CX, SH) will assess study titles and abstracts. If the title or abstract suggest that the study might meet the inclusion criteria, both reviewers will assess the full article. Differences of opinion will be resolved by group discussion (CX, SH, and TM), with the goal to reach consensus in each case. Studies failing to meet the inclusion criteria will be excluded and the reason will be reported.

Data extraction and quality assessment

Study quality will be assessed independently by two researchers (TM, CX) using guidelines for assessing prognostic studies.[29] First, two researchers will independently assess the items related to potential sources of bias, namely: (i) study participation and attrition; (ii) prognostic factor and outcome measurements; (iii) confounding measurement and account; and (iv) analyses). Then, the same two reviewers will judge the presence of potential biases as "Yes", "Partly", "No", or "Unsure". Following these steps, the Scottish Intercollegiate Guidelines Network (SIGN) [30] methodology will be implemented where "++" will be assigned to each study when all or most of the quality criteria were fulfilled (allowing one "Partly" while appraising all potential sources of bias); "+" when some of the criteria were fulfilled; and "–" when few or no criteria fulfilled (at least one "Yes"). In our review, we will refer to group "++" as 'high quality studies' and group "+" as 'moderate quality studies'. We will abstract data on the relationships between our outcomes of interest and primary (i.e., comorbidity) and secondary (i.e., socio-demographic, TBI-related) predictors only from studies with sufficient quality (i.e., 'high' and 'moderate' quality studies).[30]

Dealing with missing data

Primary authors will be contacted in cases of missing data. The proportion of missing data will be reported along with reasons where indicated. In the case of duplicate publications and

For peer review only

companion papers of a primary study, we will attempt to yield maximum scientific information by abstraction of all available data. However, original publication (usually the earliest publication version) will take priority in data analysis.

Data synthesis and analysis

For the unadjusted analysis (Step 1), we will extract data from all studies that reported the effect of comorbidity on mortality and functional status and report number of events (death and functional decline/gain/plato) relative to the total number of participants in the group with comorbid disorder(s) and control groups. The timeframe of follow- up assessments will be categorized into (i) short term, i.e., up to three months post injury mark; (ii) intermediate, i.e., 3-12 months (i.e., up to one year inclusive), and (iii) long-term, i.e., >1 year post-injury. These points were arbitrarily set. We reserve the right to adjust follow-up time frames based on time stratification applied in the included studies.

This stage will be followed by adjusted analysis (Step 2), where we will extract and analyze quantitative data (i.e., ORs, RRs, and HRs) that were adjusted for hypothesized key confounders (age, sex, TBI mechanism and severity, and/or baseline functional status) reflecting the association between comorbidity and our outcomes of interest. Some of these variables, such as age, sex, and TBI severity are considered as key confounders, and will be included in our list of required adjusted variables for a study to be included in our primary analysis.[29] If the key variables are not included in the final adjusted model, control for confounding variables will be determined to be inadequate, which will be reflected in a risk of bias assessment table.[29] Where possible, we will perform a meta-analytic analysis: pooled-effect outcomes for each group of comorbid disorders will be calculated using inverse variance methods with random-effects

For peer review only - http://bmjopen.bmj.com/site/about/guidelines.xhtml

models[31] and expressed as ORs and 95% CIs. Heterogeneity will be assessed using the I^2 statistic. P values of 0.05 or less will be considered as statistically significant.

Ethics and dissemination

This systematic review aims to investigate the relationship between comorbidity and functional outcomes and death within short, moderate and long-term time frames after TBI, as well as determining the prognostic value of comorbidity, sociodemographic (i.e., age, sex), and injury-related characteristics on these outcomes. The strength of this systematic review and research program is in its methodology, making it possible to identify associations longitudinally, thus improving the quality of inductive inferences regarding the natural progression of associative values of hypothesized predictors on outcomes in patients with TBI of various severities. Furthermore, our protocol was registered PROSPERO and was designed in keeping with best-practice methods where the multilevel risk of bias assessment[32] will allow us to detect the main flaws in the individual studies' design and inform on the future research of comorbidities in TBI. Moreover, the clinical criteria for the diagnoses of TBI and comorbidity will be collected and reported, as it is expected that they have a significant impact on the study results. Finally, multi-level knowledge translation activities throughout this research activity will be performed, ensuring that these results reach their intended knowledge users.

Limitations

The present study includes the following limitations: (1) the limiter of six months for zero-time may not be optimal as some comorbid disorder (demyelinating, degenerative, etc.) may take longer time to develop ; (2) the assumption of expected heterogeneity in the primary studies with respect to TBI-related characteristics (i.e., injury/localization of injury, time since injury) with

For peer review only - http://bmjopen.bmj.com/site/about/guidelines.xhtml

severe TBI cases expect to be underrepresented; likewise severe comorbid disorders may precluded TBI patients with milder severities of injury to participate in research, limiting the precision of estimates of risk for severe TBI and comorbidity cases; (3) potentially unequal sex and age distribution in primary studies, given that historically TBI has been considered an injury of younger males and older females; (4) residual confounding due to TBI- or comorbidity-related treatment effect; (5) excepted complexity of assessing the risk of short-term functional change (i.e., fluctuation); and (6) additional limitations relate to the exclusion of grey literature, non-English language articles, and unpublished manuscripts and their potentially relevant results; the decision was based on the extensive number of studies identified within the databases searched, as well as limited empiric evidence about the potential impact of selective searching and inclusion of these works on the results of systematic reviews.[32]

Despite these limitations, this protocol is for a review, that is the first that comprehensively synthesizes evidence on prognostic value of comorbidity in TBI patients, aiming to enrich science and advance care provided to patients with comorbid disorders stemming from traumatic brain injuries.

Implications

The number of people surviving TBIs is increasing. While the neurological consequences of TBI are well described, evidence is emerging on associations between brain injury, comorbid disorders and adverse short and long-term outcomes post-injury.[33,34] The significant economic and human costs of TBIs merit the call for systematic efforts to understand all factors that contribute to adverse post-injury outcomes, including comorbidity;[35] all with the goal to allow better risk stratification to guide management of brain injury acutely and at the chronic stages post injury on a population level.

For peer review only - http://bmjopen.bmj.com/site/about/guidelines.xhtml

Funding sources

The study was funded by the National Institutes of Health (NIH) under Award 1R21 HD08106-01 (PI: Dr. Angela Colantonio). AC is supported by the Canadian Institutes for Health Research Grant–Institute for Gender and Health (#CGW-126580). TM is supported by the postdoctoral research grant Alzheimer's Association (AARF-16-442937). The funders had no role in study design, data collection, decision to publish, or preparation of the manuscript.

Competing interests

The authors have no conflict of interest to declare pertaining to this review.

Authors' contributions

Protocol concept and design: Tatyana Mollayeva, Angela Colantonio. Registry PROSPERO: Chen Xiong. Acquisition of data: Tatyana Mollayeva. Administrative, technical, and material support: Chen Xiong, Sara Hanafy. Statistical analysis approach: Vincy Chan, Zheng Jing Hu, Mitchell Sutton, Michael Escobar. Drafting of the manuscript: Tatyana Mollayeva. Critical revision of the manuscript for important intellectual content: All authors.

Human and Animal Rights and Informed Consent

This article does not contain any studies with human or animal subjects performed by any of the authors.

Acknowledgments

We gratefully acknowledge the involvement of Ms. Jessica Babineau, Information Specialist at the Toronto Rehabilitation Institute- University Health Network for her comprehensive literature search.

For peer review only

Additional material

File name: Supplementary TableS1.Search details

File format: Table S1_10072017.pdf

Title of data: Search strategies.

Description of data: This file provides the list of search terms used to search Medline, Central, Embase, Scopus, PsycINFO, and supplemental PubMed.

For peer review only

References

1. Brain Injury Association of America. About Brain Injury. Retrieved June12, 2017, from http://www.biausa.org/about-brain-injury.htm.
2. World Health Organization. 2006. Projections of Mortality and Burden of Disease, 2002−2030. Deaths by Income Group. [Computer file] Retrieved June 12, 2017, from <www.who.int/healthinfo/global_burden_disease/projections2002/en/>.
3. Coronado VG, McGuire LC, Sarmiento K, Bell J, Lionbarger MR, Jones CD, Geller AI, Khoury N, Xu L. Trends in Traumatic Brain Injury in the U.S. and the public health response: 1995-2009. *J Safety Res.* 2012;43(4):299-307.
4. Public Health Agency of Canada. 2014. Mapping Connections: An Understanding of Neurological Conditions in Canada. Ottawa, ON: Public Health Agency of Canada. Retrieved June 12, 2017, from <www.phac-aspc.gc.ca/publicat/cd-mc/mc-ec/assets/pdf/mc-ec-eng.pdf>.
5. Colantonio A, Saverino C, Zagorski B, Swaine B, Lewko J, Jaglal S et al. Hospitalizations and Emergency Department Visits for TBI in Ontario. *Can J Neuroll Sci*. 2010; 37(6): 783–90.
6. Feinstein AR. The pre-therapeutic classification of co-morbidity in chronic disease. *J Chron Disease.* 1970; 23: 455–68.
7. Trevena L, Cameron I. Traumatic brain injury - long term care of patients in general practice. *Aust Fam Physician.* 2011;40(12):956-61.
8. Brooks JC, Shavelle RM, Strauss DJ, Hammond FM, Harrison-Felix CL. Long-Term Survival After Traumatic Brain Injury Part II: Life Expectancy. *Arch Phys Med Rehabil.* 2015; 96(6):1000-5.

For peer review only

9. Fu TS, Jing R, McFaull SR, Cusimano MD. Recent trends in hospitalization and in-hospital mortality associated with traumatic brain injury in Canada: A nationwide, population-based study. *J Trauma Acute Care Surg.* 2015; 79(3 Suppl 1):449-55.

10. Popescu C, Anghelescu A, Daia C, Onose G. Actual data on epidemiological evolution and prevention endeavours regarding traumatic brain injury. *J Med Life.* 2015;8(3):272-7.

11. Valderas JM, Starfiels B, Sibbald B, Salisbury C, Roland M. Defining comorbidity: implications for understanding health and health services. *Ann Fam Med*. 2009; 7:357-63.

12. Gardizi E, Hanks RA, Millis SR, Figueroa MJ. Comorbidity and insurance as predictors of disability following traumatic brain injury. *Arch Phys Med Rehabil.* 2014; 95:2396–401.

13. Coronado VG, Thomas KE, Sattin RW, Johnson RL. The CDC traumatic brain injury surveillance system: Characteristics of persons aged 65 years and older hospitalized with a TBI. *J Head Trauma Rehabil.* 2005; 20(3): 215–28.

14. Colantonio A, Gerber G, Bayley M, Deber R, Yin J, Kim H. Differential profiles for patients with traumatic and non-traumatic brain injury. *J Rehabil Med.* 2011; 43(4): 311–15

15. Thompson HJ, Dikmen S, Temkin N. Prevalence of comorbidity and its association with traumatic brain injury and outcomes in older adults. *Res Gerontol Nurs.* 2012; 5(1): 17–24

16. Gardizi E, Hanks RA, Millis SR, Figueroa MJ. Comorbidity and insurance as predictors of disability following traumatic brain injury. *Arch Phys Med Rehabil.* 2014; 95(12): 2396–01.

For peer review only

17. Kumar RG, Juengst SB, Wang Z, Dams-O'Connor K, Dikmen SS, O'Neil-Pirozzi TM, Dahdah MN, Hammond FM, Felix ER, Arenth PM, Wagner AK. Epidemiology of Comorbid Conditions Among Adults 50 Years and Older With Traumatic Brain Injury. *J Head Trauma Rehabil.* 2017 [Epub ahead of print] doi: 10.1097/HTR.0000000000000273.

18. Fu TS, Jing R, McFaull SR, Cusimano MD. Recent trends in hospitalization and in-hospital mortality associated with traumatic brain injury in Canada: A nationwide, population-based study. *J Trauma Acute Care Surg.* 2015; 79(3):449-54.

19. Ventura T, Harrison-Felix C, Carlson N, Diguiseppi C, Gabella B, Brown A, Devivo M, Whiteneck G. Mortality after discharge from acute care hospitalization with traumatic brain injury: a population-based study. *Arch Phys Med Rehabil.* 2010;91(1):20-9.

20. Hodgkinson A, Veerabangsa A, Drane D, McCluskey A. Service utilization following traumatic brain injury. *J Head Trauma Rehabil.* 2000;15(6):1208-26.

21. Perrin PB, Niemeier JP, Mougeot JL, Vannoy CH, Hirsch MA, Watts JA, Rossman W, Grafton LM, Guerrier TD, Pershad R, Kingsbury CA, Bartel SW, Whitney MP. Measures of injury severity and prediction of acute traumatic brain injury outcomes. *J Head Trauma Rehabil.* 2015; 30(2):136-42.

22. Krasny-Pacini A, Chevignard M, Lancien S, Escolano S, Laurent-Vannier A, De Agostini M, Meyer P. Executive function after severe childhood traumatic brain injury - Age-at-injury vulnerability periods: The TGE prospective longitudinal study. *Ann Phys Rehabil Med.* 2017;60(2):74-82.

23. Klauber MR, Marshall LF, Barrett-Connor E, et al: Prospective study of patients hospitalized with head injury in San Diego County, 1978. *Neurosurgery.* 1981; 9:236–41.

For peer review only

24. Slewa-Younan S, van den Berg S, Baguley IJ, Nott M, Cameron ID. Towards an understanding of sex differences in functional outcome following moderate to severe traumatic brain injury: a systematic review. *J Neurol Neurosurg Psychiatry*. 2008;79(11):1197-201.

25. Moher D, Liberati A, Tetzlaff J, Altman DG. Preferred reporting items for systematic reviews and meta-analyses for PRISMA statement. *PLoS Medicine.* 2009; 6(7).

26. PROSPERO- International Prospective Register of Systematic Reviews. [http://www.crd.york.ac.uk/prospero/].

27. Valderas JM, Starfiels B, Sibbald B, Salisbury C, Roland M. Defining comorbidity: implications for understanding health and health services. *Ann Fam Med.* 2009; 7:357-363.

28. Mollayeva T, Kendzerska T, Mollayeva S, Shapiro CM, Colantonio A, Cassidy JD. A systematic review of fatigue in patients with traumatic brain injury: the course, predictors and consequences. *Neurosci Biobehav Rev.* 2014;47: 684-716.

29. Hayden JA, Cote P, Bombardier C. Evaluation of the quality of prognosis studies in systematic reviews. *Ann Intern Med.* 2006; 144: 427-37.

30. Scottish Intercollegiate Guidelines Network: Published Guidelines. Retrieved 12 06 2017, from http://www.sign.ac.uk.guidelines/

31. Hardy RJ, Thompson SG. Detecting and describing heterogeneity in meta-analysis. *Stat Med.* 1998; 17(8):841-56.

32. Hartling L, Featherstone R, Nuspl M, Shave K, Dryden DM, Vandermeer B. Grey literature in systematic reviews: a cross-sectional study of the contribution of non-English

For peer review only

reports, unpublished studies and dissertations to the results of meta-analyses in child-relevant reviews. Reference: *BMC Med Res Methodol.* 2017;17(1):64.

33. Shukla D, Devi BI, Agrawal A. Outcome measures for traumatic brain injury. *Clin Neurol Neurosurg.* 2011; 113(6):435-41.

34. Nichol AD, Higgins AM, Gabbe BJ, Murray LJ, Cooper DJ, Cameron PA. Measuring functional and quality of life outcomes following major head injury: common scales and checklists. *Injury.* 2011;42(3):281-7.

35. Charlson ME, Pompei P, Ales KL, MacKenzie CR. A new method of classifying prognostic comorbidity in longitudinal studies: development and validation. *J Chronic Dis.* 1987;40(5):373-83.

For peer review only

Table 1. Prognosis, traumatic brain injury (TBI) and outcomes-related search terms (from Medline search strategy).

**Prognosis terms**	Exp cohort studies/ or exp prognosis/ or exp morbidity/ or exp mortality/ or exp survival analysis/ or exp models, statistical/ or prognos*.tw./ or predict*.tw./ or course*.tw./ or diagnosed.tw./ or cohort*.tw./ or death.tw./ or exp treatment outcome/ or "early termination of clinical trials"/ or treatment failure/ incidence/
**TBI terms**	Exp brain injuries/ or Craniocerebral Trauma/ or exp Head Injuries, Closed/ or exp Skull Fractures/ or mTBI*2.tw,kw. or tbi*2.tw,kw. or concuss*.tw,kw. or ((head* or cerebr* or crani* or skull* or intracran*) adj2 (injur* or trauma* or damag* or wound* or swell* or oedema* or edema* or fracture* or contusion* or pressur*)).tw,kw. or ((brain* or cerebr* or intracerebr* or crani* or intracran* or head* or subdural* or epidural* or extradural*) adj (haematoma* or hematoma* or hemorrhag* or haemorrhag* or bleed*)).tw,kw.
**Comorbidity terms**	Exp Comorbidity/ or exp Risk Adjustment/ or (comorbid* or co morbid* or multimorbid* or multi morbid*).tw,kw. or (polypatholog* or poly-patholog*).tw,kw. or ((clinical* or medical*) adj3 complex*).tw,kw. or ((coexist* or co exist* or cooccur* or co-occur* or multipl*) adj3 (illness* or disease* or disorder* or condition* or complication* or diagnos* or risk*)).tw,kw. or (multidisease? or multi-disease? or (multiple adj (ill* or disease? or condition? or syndrom* or disorder?))).tw,kw. or ((several* or various or (two adj2 more) or concomitant or conjoined or concurrent) adj3 (morbid* or ill* or disease* or sick* or condition*)).tw,kw. Or "comorbidity-polypharmacy score".tw,kw. or ('charlson comorbidity index' or 'CCI' or 'CMI' or elixhauser or 'BOD index' or 'cumulative index rating scale' or 'CIRS' or 'Coroni-Huntley index' or 'DUSOI index' or 'Hallstrom index' or 'Hurwitz index' or 'Incalzi index', 'Kaplan index', 'Liu index', 'Shwartz index' or 'comorbidity-polypharmacy score').tw,kw.
**Outcomes terms**	Exp Mortality/ or exp morbidity/ or (morbidit* or mortalit*).tw,kw. or function*.mp.

For peer review only

For peer review only - http://bmjopen.bmj.com/site/about/guidelines.xhtml

Supplementary Table S1. Search details.
Searches conducted in Medline (including Medline in Process and other non-indexed citations, ePubs and Medline Daily), Embase, Cochrane Central Register of Controlled Trials, and PsycINFO. Searches were limited from 1997 to May 2017. Searches were limited to English language papers, and to an adult, human population when possible. Searches were conducted by an Information Specialist (JB).

PRE-DUPLICATE REMOVAL
TOTAL Results: 9414
Medline: 2860
Central: 178
Embase: 5468
PsycINFO: 908

POST-DUPLICATE REMOVAL
TOTAL Results: 7443
Duplicates removed: 1971
Medline: 2748
Central: 116
Embase: 4088
PsycINFO: 491

Database: Ovid MEDLINE(R) Epub Ahead of Print, In-Process & Other Non-Indexed Citations, Ovid MEDLINE(R) Daily and Ovid MEDLINE(R) <1946 to Present>
Search Strategy:
----
1 exp brain injuries/ (59169)
2 Craniocerebral Trauma/ (21199)
3 exp Head Injuries, Closed/ (9232)
4 exp Skull Fractures/ (20700)
5 mTBI*2.tw,kw. (1835)
6 tbi*2.tw,kw. (20636)
7 concuss*.tw,kw. (6496)
8 ((head* or cerebr* or crani* or skull* or intracran*) adj2 (injur* or trauma* or damag* or wound* or swell* or oedema* or edema* or fracture* or contusion* or pressur*)).tw,kw. (75791)
9 ((brain* or cerebr* or intracerebr* or crani* or intracran* or head* or subdural* or epidural* or extradural*) adj (haematoma* or hematoma* or hemorrhag* or haemorrhag* or bleed*)).tw,kw. (42638)
10 or/1-9 (185742)
11 exp Comorbidity/ (88810)
12 exp Risk Adjustment/ (2770)
13 (comorbid* or co morbid* or multimorbid* or multi morbid*).tw,kw. (131913)
14 (polypatholog* or poly-patholog*).tw,kw. (151)
15 ((clinical* or medical*) adj3 complex*).tw,kw. (11314)

For peer review only

16 ((coexist* or co exist* or cooccur* or co-occur* or multipl*) adj3 (illness* or disease* or disorder* or condition* or complication* or diagnos* or risk*)).tw,kw. (69114)
17 (multidisease? or multi-disease? or (multiple adj (ill* or disease? or condition? or syndrom* or disorder?))).tw,kw. (3721)
18 ((several* or various or (two adj2 more) or concomitant or conjoined or concurrent) adj3 (morbid* or ill* or disease* or sick* or condition*)).tw,kw. (129147)
19 "comorbidity-polypharmacy score".tw,kw. (11)
20 ('charlson comorbidity index' or 'CCI' or 'CMI' or elixhauser or 'BOD index' or 'cumulative index rating scale' or 'CIRS' or 'Coroni-Huntley index' or 'DUSOI index' or 'Hallstrom index' or 'Hurwitz index' or 'Incalzi index', 'Kaplan index', 'Liu index', 'Shwartz index' or 'comorbidity-polypharmacy score').tw,kw. (10394)
21 or/11-20 (394772)
22 10 and 21 (5122)
23 exp Mortality/ (335478)
24 exp morbidity/ (467773)
25 (morbidit* or mortalit*).tw,kw. (760891)
26 function*.mp. (3282516)
27 or/23-26 (4495784)
28 exp cohort studies/ (1680971)
29 exp prognosis/ (1379668)
30 exp survival analysis/ (241795)
31 exp models, statistical/ (344993)
32 prognos*.tw,kw. (492733)
33 predict*.tw,kw. (1261259)
34 course*.tw,kw. (546731)
35 diagnosed.tw,kw. (461120)
36 cohort*.tw,kw. (423751)
37 death.tw,kw. (584394)
38 exp treatment outcome/ (841041)
39 "early termination of clinical trials"/ (544)
40 treatment failure/ (31446)
41 incidence/ (222806)
42 or/28-41 (5055925)
43 27 or 42 (8021818)
44 22 and 43 (3756)
45 44 not (exp animals/ not exp humans/) (3301)
46 45 not (exp children/ not exp adults/) (3118)
47 limit 46 to english language (2860)
48 remove duplicates from 47 (2750)

****
Database: Embase <1974 to 2017 May 10>
Search Strategy:
----
1 exp brain injury/ (148571)

For peer review only - http://bmjopen.bmj.com/site/about/guidelines.xhtml

2 head injury/ (44023)
3 mTBI*2.tw. (2763)
4 tbi*2.tw. (32342)
5 concuss*.tw. (7543)
6 ((head* or cerebr* or crani* or skull* or intracran*) adj2 (injur* or trauma* or damag* or wound* or swell* or oedema* or edema* or fracture* or contusion* or pressur*)).tw,kw. (96428)
7 ((brain* or cerebr* or intracerebr* or crani* or intracran* or head* or subdural* or epidural* or extradural*) adj (haematoma* or hematoma* or hemorrhag* or haemorrhag* or bleed*)).tw,kw. (59251)
8 or/1-7 (291625)
9 comorbidity/ (177225)
10 risk assessment/ (409214)
11 exp comorbidity assessment/ (9540)
12 (comorbid* or co morbid* or multimorbid* or multi morbid*).tw,kw. (217271)
13 (polypatholog* or poly-patholog*).tw,kw. (290)
14 ((clinical* or medical*) adj3 complex*).tw,kw. (15500)
15 ((coexist* or co exist* or cooccur* or co-occur* or multipl*) adj3 (illness* or disease* or disorder* or condition* or complication* or diagnos* or risk*)).tw,kw. (94039)
16 (multidisease? or multi-disease? or (multiple adj (ill* or disease? or condition? or syndrom* or disorder?))).tw,kw. (4710)
17 ((several* or various or (two adj2 more) or concomitant or conjoined or concurrent) adj3 (morbid* or ill* or disease* or sick* or condition*)).tw,kw. (163497)
18 "comorbidity-polypharmacy score".tw,kw. (15)
19 ('charlson comorbidity index' or 'CCI' or 'CMI' or elixhauser or 'BOD index' or 'cumulative index rating scale' or 'CIRS' or 'Coroni-Huntley index' or 'DUSOI index' or 'Hallstrom index' or 'Hurwitz index' or 'Incalzi index', 'Kaplan index', 'Liu index', 'Shwartz index' or 'comorbidity-polypharmacy score').tw,kw. (19018)
20 or/9-19 (943533)
21 8 and 20 (15388)
22 exp mortality/ (858109)
23 exp morbidity/ (299827)
24 (morbidit* or mortalit*).tw,kw. (1038507)
25 function*.mp. (4279406)
26 or/22-25 (5472712)
27 cohort analysis/ (287239)
28 prognosis/ (506894)
29 survival analysis/ (3712)
30 exp survival/ (845725)
31 statistical model/ (136685)
32 exp "prediction and forecasting"/ (1010760)
33 prognos*.tw,kw. (700803)
34 predict*.tw,kw. (1580756)
35 course*.tw,kw. (702204)
36 diagnosed.tw,kw. (688998)

For peer review only

For peer review only - http://bmjopen.bmj.com/site/about/guidelines.xhtml

37 cohort*.tw,kw. (649460)
38 death.tw,kw. (778611)
39 exp treatment outcome/ (1236966)
40 "early termination of clinical trial"/ (204)
41 exp treatment failure/ (108708)
42 exp incidence/ (330423)
43 or/27-42 (5901375)
44 26 or 43 (9681606)
45 21 and 44 (10931)
46 45 not ((exp animals/ or exp animal experimentation/ or nonhuman/) not exp human/) (9877)
47 46 not ((exp embryo/ or exp fetus/ or exp juvenile/) not exp adult/) (8968)
48 limit 47 to english language (8463)
49 limit 48 to embase (5871)
50 limit 49 to (conference abstract or conference paper or "conference review") (179)
51 49 not 50 (5692)
52 limit 51 to yr="1997 -Current" (5468)

****

Database: EBM Reviews - Cochrane Central Register of Controlled Trials <April 2017>
Search Strategy:
----
1 exp brain injuries/ (1135)
2 Craniocerebral Trauma/ (247)
3 exp Head Injuries, Closed/ (167)
4 exp Skull Fractures/ (192)
5 mTBI*2.tw,kw. (122)
6 tbi*2.tw,kw. (1306)
7 concuss*.tw,kw. (221)
8 ((head* or cerebr* or crani* or skull* or intracran*) adj2 (injur* or trauma* or damag* or wound* or swell* or oedema* or edema* or fracture* or contusion* or pressur*)).tw,kw. (3278)
9 ((brain* or cerebr* or intracerebr* or crani* or intracran* or head* or subdural* or epidural* or extradural*) adj (haematoma* or hematoma* or hemorrhag* or haemorrhag* or bleed*)).tw,kw. (3605)
10 or/1-9 (8539)
11 exp Comorbidity/ (3012)
12 exp Risk Adjustment/ (20)
13 (comorbid* or co morbid* or multimorbid* or multi morbid*).tw,kw. (9750)
14 (polypatholog* or poly-patholog*).tw,kw. (4)
15 ((clinical* or medical*) adj3 complex*).tw,kw. (711)
16 ((coexist* or co exist* or cooccur* or co-occur* or multipl*) adj3 (illness* or disease* or disorder* or condition* or complication* or diagnos* or risk*)).tw,kw. (4140)
17 (multidisease? or multi-disease? or (multiple adj (ill* or disease? or condition? or syndrom* or disorder?))).tw,kw. (107)

For peer review only - http://bmjopen.bmj.com/site/about/guidelines.xhtml
For peer review only

18 ((several* or various or (two adj2 more) or concomitant or conjoined or concurrent) adj3 (morbid* or ill* or disease* or sick* or condition*)).tw,kw. (1145)
19 "comorbidity-polypharmacy score".tw,kw. (0)
20 ('charlson comorbidity index' or 'CCI' or 'CMI' or elixhauser or 'BOD index' or 'cumulative index rating scale' or 'CIRS' or 'Coroni-Huntley index' or 'DUSOI index' or 'Hallstrom index' or 'Hurwitz index' or 'Incalzi index', 'Kaplan index', 'Liu index', 'Shwartz index' or 'comorbidity-polypharmacy score').tw,kw. (748)
21 or/11-20 (17592)
22 10 and 21 (214)
23 22 not (exp animals/ not exp humans/) (214)
24 23 not (exp Child/ not exp Adult/) (213)
25 limit 24 to english language (185)
26 limit 25 to yr="1997 -Current" (178)

****

Database: PsycINFO <1806 to May Week 1 2017>
Search Strategy:

----

1 exp traumatic brain injury/ (15677)
2 exp head injuries/ (5584)
3 mTBI*2.tw. (1312)
4 tbi*2.tw. (8651)
5 concuss*.tw. (2360)
6 ((head* or cerebr* or crani* or skull* or intracran*) adj2 (injur* or trauma* or damag* or wound* or swell* or oedema* or edema* or fracture* or contusion* or pressur*)).tw. (11318)
7 ((brain* or cerebr* or intracerebr* or crani* or intracran* or head* or subdural* or epidural* or extradural*) adj (haematoma* or hematoma* or hemorrhag* or haemorrhag* or bleed*)).tw. (3029)
8 or/1-7 (28382)
9 comorbidity/ (27127)
10 risk assessment/ (11895)
11 (comorbid* or co morbid* or multimorbid* or multi morbid*).tw. (49497)
12 (polypatholog* or poly-patholog*).tw. (23)
13 ((clinical* or medical*) adj3 complex*).tw. (2577)
14 ((coexist* or co exist* or cooccur* or co-occur* or multipl*) adj3 (illness* or disease* or disorder* or condition* or complication* or diagnos* or risk*)).tw. (16826)
15 (multidisease? or multi-disease? or (multiple adj (ill* or disease? or condition? or syndrom* or disorder?))).tw. (666)
16 ((several* or various or (two adj2 more) or concomitant or conjoined or concurrent) adj3 (morbid* or ill* or disease* or sick* or condition*)).tw. (14708)
17 "comorbidity-polypharmacy score".tw. (2)
18 ('charlson comorbidity index' or 'CCI' or 'CMI' or elixhauser or 'BOD index' or 'cumulative index rating scale' or 'CIRS' or 'Coroni-Huntley index' or 'DUSOI index' or

For peer review only

'Hallstrom index' or 'Hurwitz index' or 'Incalzi index', 'Kaplan index', 'Liu index', 'Shwartz index' or 'comorbidity-polypharmacy score').tw,tm. (2701)
19 or/9-18 (97253)
20 8 and 19 (1354)
21 limit 20 to animal (317)
22 limit 20 to human (1042)
23 20 not (21 not 22) (1071)
24 limit 23 to ((childhood <birth to 12 years> or adolescence <13 to 17 years>) and (100 childhood <birth to age 12 yrs> or 120 neonatal <birth to age 1 mo> or 140 infancy <2 to 23 mo> or 160 preschool age <age 2 to 5 yrs> or 180 school age <age 6 to 12 yrs> or 200 adolescence <age 13 to 17 yrs>)) (132)
25 limit 23 to (adulthood <18+ years> and ("300 adulthood <age 18 yrs and older>" or 320 young adulthood <age 18 to 29 yrs> or 340 thirties <age 30 to 39 yrs> or 360 middle age <age 40 to 64 yrs> or "380 aged <age 65 yrs and older>" or "390 very old <age 85 yrs and older>")) (589)
26 23 not (24 not 25) (1023)
27 limit 26 to english language (977)
28 limit 27 to yr="1997 -Current" (908)

****

For peer review only

For peer review only - http://bmjopen.bmj.com/site/about/guidelines.xhtml

PRISMA-P (Preferred Reporting Items for Systematic review and Meta-Analysis Protocols) 2015 checklist: recommended items to address in a systematic review protocol*

Section and topic	Item No	Checklist item	Location; page(s) within the manuscript
**ADMINISTRATIVE INFORMATION**			
Title:			
Identification	1a	Identify the report as a protocol of a systematic review	Title, abstract, manuscript; pgs. 1-18
Update	1b	If the protocol is for an update of a previous systematic review, identify as such	NA; original review protocol
Registration	2	If registered, provide the name of the registry (such as PROSPERO) and registration number	PROSPERO: CRD42017070033
Authors:			
Contact	3a	Provide name, institutional affiliation, e-mail address of all protocol authors; provide physical mailing address of corresponding author	Title page: 1-2
Contributions	3b	Describe contributions of protocol authors and identify the guarantor of the review	Pgs.18
Amendments	4	If the protocol represents an amendment of a previously completed or published protocol, identify as such and list changes; otherwise, state plan for documenting important protocol amendments	N/A
Support:			
Sources	5a	Indicate sources of financial or other support for the review	Pg. 17
Sponsor	5b	Provide name for the review funder and/or sponsor	Pg. 17
Role of sponsor or funder	5c	Describe roles of funder(s), sponsor(s), and/or institution(s), if any, in developing the protocol	Pg. 17
**INTRODUCTION**			
Rationale	6	Describe the rationale for the review in the context of what is already known	Pgs. 8-9
Objectives	7	Provide an explicit statement of the question(s) the review will address with reference to participants, interventions, comparators, and outcomes (PICO)	Pg. 9
**METHODS**			

For peer review only

Eligibility criteria	8	Specify the study characteristics (such as PICO, study design, setting, time frame) and report characteristics (such as years considered, language, publication status) to be used as criteria for eligibility for the review	Pg. 12
Information sources	9	Describe all intended information sources (such as electronic databases, contact with study authors, trial registers or other grey literature sources) with planned dates of coverage	Pg. 12
Search strategy	10	Present draft of search strategy to be used for at least one electronic database, including planned limits, such that it could be repeated	Supplementary file Table S1
Study records:			
Data management	11a	Describe the mechanism(s) that will be used to manage records and data throughout the review	Pg. 13-14
Selection process	11b	State the process that will be used for selecting studies (such as two independent reviewers) through each phase of the review (that is, screening, eligibility and inclusion in meta-analysis)	Pg. 13
Data collection process	11c	Describe planned method of extracting data from reports (such as piloting forms, done independently, in duplicate), any processes for obtaining and confirming data from investigators	Pg. 13-14
Data items	12	List and define all variables for which data will be sought (such as PICO items, funding sources), any pre-planned data assumptions and simplifications	Pgs.11-12
Outcomes and prioritization	13	List and define all outcomes for which data will be sought, including prioritization of main and additional outcomes, with rationale	Pg. 11
Risk of bias in individual studies	14	Describe anticipated methods for assessing risk of bias of individual studies, including whether this will be done at the outcome or study level, or both; state how this information will be used in data synthesis	Pgs.13-14
Data synthesis	15a	Describe criteria under which study data will be quantitatively synthesised	Pg. 14-15
	15b	If data are appropriate for quantitative synthesis, describe planned summary measures, methods of handling data and methods of combining data from studies, including any planned exploration of consistency (such as I^2, Kendall's τ)	Pg. 14
	15c	Describe any proposed additional analyses (such as sensitivity or subgroup analyses, meta-regression)	Pg. 15
	15d	If quantitative synthesis is not appropriate, describe the type of summary planned	N/A

For peer review only

Meta-bias(es)	16	Specify any planned assessment of meta-bias(es) (such as publication bias across studies, selective reporting within studies)	N/A
Confidence in cumulative evidence	17	Describe how the strength of the body of evidence will be assessed (such as GRADE)	Pg. 13-14

*** It is strongly recommended that this checklist be read in conjunction with the PRISMA-P Explanation and Elaboration (cite when available) for important clarification on the items. Amendments to a review protocol should be tracked and dated. The copyright for PRISMA-P (including checklist) is held by the PRISMA-P Group and is distributed under a Creative Commons Attribution Licence 4.0.**

From: Shamseer L, Moher D, Clarke M, Ghersi D, Liberati A, Petticrew M, Shekelle P, Stewart L, PRISMA-P Group. Preferred reporting items for systematic review and meta-analysis protocols (PRISMA-P) 2015: elaboration and explanation. BMJ. 2015 Jan 2;349(jan02 1):g7647.

For peer review only

BMJ Open

Comorbidity and outcomes in traumatic brain injury: Protocol for a systematic review on functional status and risk of death.

Journal:	*BMJ Open*
Manuscript ID	bmjopen-2017-018626.R1
Article Type:	Protocol
Date Submitted by the Author:	04-Sep-2017

Complete List of Authors:	Mollayeva , Tatyana; Toronto Rehabilitation Institute, Research; University of Toronto, Rehabilitation Sciences Institute<br>Xiong, Chen; Rehabilitation Sciences Institute , University of Toronto<br>Hanafy, Sara; Toronto Rehabilitation Institute-University Health Network, Research<br>Chan , Vincy ; Toronto Rehabilitation Institute , Research<br>Hu, Zheng Jing ; Toronto Rehabilitation Institute-University Health Network, Research<br>Sutton, Mitchell; Toronto Rehabilitation Institute-University Health Network, Research<br>Escobar, Michael ; University of Toronto Dalla Lana School of Public Health<br>Colantonio, Angela; University of Toronto, Rehabilitation Science Institute

**Primary Subject Heading**:	Epidemiology
Secondary Subject Heading:	Research methods, Neurology
Keywords:	comorbidity, traumatic brain injury, prognostic review, mortality, cognitive/physical status, sex and age stratification

SCHOLARONE™
Manuscripts

For peer review only

TITLE: Comorbidity and outcomes in traumatic brain injury: Protocol for a systematic review on functional status and risk of death.

Authors: Tatyana Mollayeva, MD, PhD [1-3], Chen Xiong, MSc[1-3], Sara Hanafy, MSc[2,3], Vincy Chan, MPH, PhD[2-4], Zheng Jing Hu, MSc[5], Mitchell Sutton, MSc[5], Michael Escobar, PhD[5], Angela Colantonio, PhD [1-3,5]

Author details

[1]Rehabilitation Sciences Institute, Faculty of Medicine, University of Toronto, Canada

[2]Toronto Rehabilitation Institute-University Health Network, Canada

[3]Aquired Brain Injury Research Lab, University of Toronto, Canada

[4] Pediatric Oncology Group of Ontario, Canada

[5]Dalla Lana School of Public Health, University of Toronto

Tatyana Mollayeva, MD, PhD (corresponding author)

Rehabilitation Sciences Institute

University of Toronto

Toronto Rehabilitation Institute-University Health Network

550 University Avenue, Rm 11207

Toronto, Ontario M5G 2A2

tatyana.mollayeva@utoronto.ca

tel: 416-597-3422 ext 7848

fax: 416-946-8570

Chen Xiong, MSc, PhD student

For peer review only

Rehabilitation Sciences Institute

Rehabilitation Sciences Building, University of Toronto

500 University Avenue, Room 260

Toronto, ON M5G 1V7

chen.xiong@mail.utoronto.ca

tel: 647-770-8366

fax: 416-946-8570

Sara Hanafy, MSc

Researcher-volunteer

Toronto Rehabilitation Institute-University Health Network

550 University Avenue

Toronto, ON M5G 2A2

sara.hanafy@utoronto.ca

tel: 416-597-3422

fax: 416-946-8570

Vincy Chan, PhD, MPH

Toronto Rehabilitation Institute-University Health Network

550 University Avenue

Toronto, ON, M5G 2A2

vincy.chan@uhn.ca

416-597-3422 ext. 7845

For peer review only

Zheng Jing Hu, MSc

Summer research intern

Toronto Rehabilitation Institute-University Health Network

550 University Avenue, Rm 11-207

Toronto, Ontario M5G 2A2

Dalla Lana School of Public Health

University of Toronto

Zhengjing.hu@mail.utoronto.ca

tel: 416-597-3422

fax: 416-946-8570

Mitchell Sutton, MSc

Summer research intern

Toronto Rehabilitation Institute-University Health Network

550 University Avenue, Rm 11-207

Toronto, Ontario M5G 2A2

Dalla Lana School of Public Health

University of Toronto

mitchell.sutton@mail.utoronto.ca

tel: 416-597-3422

fax: 416-946-8570

For peer review only

Michael Escobar, PhD

Professor, Dalla Lana School of Public Health

Health Sciences Building

155 College Street, 6th Floor

Toronto, ON M5T 3M7

m.escobar@utoronto.ca

tel: 416- 978-7942

fax: 416-946-8570

Angela Colantonio, PhD

Director, Rehabilitation Sciences Institute

University of Toronto

Senior Scientist, Toronto Rehabilitation Institute-University Health Network

160-500 University Ave.

Toronto, Ontario M5G 1V7

angela.colantonio@utoronto.ca

tel: 416-978-1098

fax: 416-946-8570

For peer review only

ABSTRACT

Introduction: Reports on the association between comorbidity and functional status and risk of death in patients with traumatic brain injury (TBI) have been inconsistent; it is currently unknown which additional clinical entities (comorbidities) have an adverse influence on the evolution of outcomes across the lifespan of males and females with TBI. The current protocol outlines a strategy for a systematic review of the current evidence examining the impact of comorbidity on functional status and early and late-term mortality, taking into account known risk factors of these adverse outcomes (i.e., demographic (age and sex), and injury-related characteristics).

Methods and analysis: A comprehensive search strategy for TBI prognosis, functional (cognitive and physical) status and mortality studies has been developed in collaboration with a medical information specialist of the large rehabilitation teaching hospital. All peer-reviewed English language studies with longitudinal design in adults with TBI of any severity, published from May 1997 to April 2017, found through Medline, Central, Embase, Scopus, PsycINFO and bibliographies of identified articles, will be considered eligible. Study quality will be assessed using published guidelines.

Ethics and dissemination: The authors will publish findings from this review in a peer-reviewed scientific journal (s) and present the results at national and international conferences. This work aims to understand how comorbidity may contribute to adverse outcomes in TBI, to inform risk stratification of patients and guide the management of brain injury acutely and at the chronic stages post-injury on a population level. *Registration details:* CRD42017070033.

For peer review only

Keywords: comorbidity; traumatic brain injury; prognostic review; mortality; cognitive status; physical status; age; sex

For peer review only

Strengths and limitations of this study

- The study of comorbidity in traumatic brain injury (TBI) is important, as any additional clinical entity can change future life course and injury outcomes
- To date, there has been no systematic review on the topic of comorbidity in TBI as it relates to all-cause mortality and functional status post injury; the protocol outlines a strategy for a study that intends to fill the gap
- Attention to known risk factors of adverse outcomes such as sex, age, and TBI severity will permit advanced risk stratification and inform future prognostic studies
- Biases associated with unequal sex and age distribution, residual confounding due to TBI- or comorbidity-related treatment effect could not be avoided
- Systematizing prognostic data on comorbidity in TBI is essential for patients, health care providers, policymakers, and health researchers.

For peer review only

Introduction

Traumatic Brain Injury (TBI), defined as "*an alteration in brain function, or other evidence of brain pathology, caused by an external force,"* [1]is a major global health concern. According to the World Health Organization, death and disability from TBI is rising rapidly.[2] In the US, the total annual cost of TBI was estimated to be $60.43 billion US dollars.[3] In Canada, a recent report by the Public Health Agency projected that the indirect economic cost of a TBI due to working-age death and disability will increase from $7.3 billion in 2011 to $8.2 billion by 2031, far exceeding that of other common neurological conditions (e.g., epilepsy, multiple sclerosis, Alzheimer's Disease, combined, with estimated $4.8 billion in 2011 and $5.8 billion in 2031, respectively).[4] TBI may also exacerbate pre-existing disorders, or expedite the development of, additional clinical conditions in both the older and younger populations, increasing direct and indirect costs associated with TBI.[5]

Of particular importance is that the presence of a comorbidity (i.e., additional disease or illness coexisting with an index disease [6]) or multiple comorbidities in patients with TBI is common,[7-9] and is associated with high rates of hospitalizations, decreased functional status, and all-cause mortality.[7-9] Studies have also shown that comorbid disorders may alter the treatment course of patients with TBI by affecting the treatments that these patients receive in both the acute and rehabilitation setting.[7-9] Specifically, among patients with a comorbid health condition, treating or managing comorbidities are often prioritized over addressing the TBI itself .[10-12] Likewise, the presence of any chronic comorbidity in TBI may lead patients to consume a disproportionate amount of healthcare resources .[13-14] While previous research has documented number of comorbidities in patients with TBI,[15-17] it is currently unknown which comorbid disorders pre-exist in TBI across ages, develop over time, and which best predict outcomes related to

For peer review only

functional (cognitive and physical) status and early and late post-injury mortality. It is also unknown if the presence of comorbidity changes the effect of traditional TBI risk factors for these outcomes, such as TBI severity and mechanism.[18-21]

Finally, it must be highlighted that a patient's sex and age has also been shown to drive differences in early mortality and functional recovery.[22-23] Previous research highlighted that case fatality ratios are elevated in older patients (60+ years of age) compared to younger patients, with sex differences observed in individuals 20 to 39 years of age .[23] Females are also at greater risk for the development of somatic and psychiatric comorbidity and associated functional decline post injury .[24] Overall, despite the strong evidence that comorbidities lead to adverse outcomes and complications among patients with TBI, a data synthesis on this topic taking sex and age into account does not exist to date. This highlights the need to explore how age and sex associate with comorbidity risk factors in patients with TBI.

As such, this protocol is for a systematic review on the topic of comorbidity in adult patients with TBI that aims to: (1) examine the relationship between comorbid disorder(s) and change in function after TBI, and death; (2) determine the prognostic value of clinical characteristics of patients with TBI at baseline on the development of short, intermediate and long term adverse or beneficial outcome (s); and (3) review effects of comorbidity in the light of currently known risk factors of adverse outcomes (i.e., sex, age, and injury severity).

Methods and analysis

The systematic review that this protocol describes will be conducted and reported in compliance with the Preferred Reporting Items for Systematic Reviews and Meta-Analyses (PRISMA) guidelines.[25] In accordance with these guidelines, this systematic review protocol was registered

For peer review only

with the International Prospective Register of Systematic Reviews (PROSPERO) on June 22, 2017.[26]

Data sources and searches

In collaboration with TBI and rehabilitation experts, and a Medical Information Specialist, a comprehensive search strategy for prognostic studies of TBI outcomes (i.e., functional status and mortality) was developed (Table 1). All English language peer-reviewed studies published between March 1997 to April 2017, with prospective or retrospective data collection and a longitudinal design, found through Medline, Central, Embase, Scopus, PsycINFO and bibliographies of identified articles will be included. Reference lists of included studies will be reviewed to identify any additional relevant studies. Search terms for each database are presented in Table S1.

Table 1. Prognosis, traumatic brain injury (TBI) and outcomes-related search terms (from Medline search strategy).

For peer review only

**Prognosis terms**	Exp cohort studies/ or exp prognosis/ or exp morbidity/ or exp mortality/ or exp survival analysis/ or exp models, statistical/ or prognos*.tw./ or predict*.tw./ or course*.tw./ or diagnosed.tw./ or cohort*.tw./ or death.tw./ or exp treatment outcome/ or "early termination of clinical trials"/ or treatment failure/ incidence/
**TBI terms**	Exp brain injuries/ or Craniocerebral Trauma/ or exp Head Injuries, Closed/ or exp Skull Fractures/ or mTBI*2.tw,kw. or tbi*2.tw,kw. or concuss*.tw,kw. or ((head* or cerebr* or crani* or skull* or intracran*) adj2 (injur* or trauma* or damag* or wound* or swell* or oedema* or edema* or fracture* or contusion* or pressur*)).tw,kw. or ((brain* or cerebr* or intracerebr* or crani* or intracran* or head* or subdural* or epidural* or extradural*) adj (haematoma* or hematoma* or hemorrhag* or haemorrhag* or bleed*)).tw,kw.
**Comorbidity terms**	Exp Comorbidity/ or exp Risk Adjustment/ or (comorbid* or co morbid* or multimorbid* or multi morbid*).tw,kw. or (polypatholog* or poly-patholog*).tw,kw. or ((clinical* or medical*) adj3 complex*).tw,kw. or ((coexist* or co exist* or cooccur* or co-occur* or multipl*) adj3 (illness* or disease* or disorder* or condition* or complication* or diagnos* or risk*)).tw,kw. or (multidisease? or multi-disease? or (multiple adj (ill* or disease? or condition? or syndrom* or disorder?))).tw,kw. or ((several* or various or (two adj2 more) or concomitant or conjoined or concurrent) adj3 (morbid* or ill* or disease* or sick* or condition*)).tw,kw. Or "comorbidity-polypharmacy score".tw,kw. or ('charlson comorbidity index' or 'CCI' or 'CMI' or elixhauser or 'BOD index' or 'cumulative index rating scale' or 'CIRS' or 'Coroni-Huntley index' or 'DUSOI index' or 'Hallstrom index' or 'Hurwitz index' or 'Incalzi index', 'Kaplan index', 'Liu index', 'Shwartz index' or 'comorbidity-polypharmacy score').tw,kw.
**Outcomes terms**	Exp Mortality/ or exp morbidity/ or (morbidit* or mortalit*).tw,kw. or function*.mp.

For peer review only

Comorbidity definition

Feinstein defined comorbidity as "the existence or occurrence of any distinct additional entity during the clinical course of a patient who has the index disease under study".[5] More recently, Valderas et al. defined it as "any additional condition that may occur during the clinical course of a patient who has an index condition that is the focus of interest".[27] Canadian coding standards, the International Classification of Diseases Version 10 (ICD-10-CA) defines comorbidity as "a condition that coexists in addition to the most responsible diagnosis (MRDx) at the time of admission or that develops subsequently and meets at least one of the three criteria for significance": (1) requires treatment beyond maintenance of the pre-existing condition; (2) increases the length of stay (LOC) by at least 24 hours; and/or (3) significantly affects the treatment received". [28] Currently, there is no gold-standard for assessing comorbidity in TBI patients and reports of comorbidity vary widely in the published work .[18-21] In population based studies, comorbid conditions can be identified according to the International Classification of Diseases (ICD) diagnostic codes; or converted into summary comorbidity measures focused on selected conditions, such as the Charlson Comorbidity Index (CCI), Aggregated Diagnosis Groups (ADGs), Elixhauser Comorbidity Index, or the Total Illness Burden Index .[18-21] However, many papers focus on a single count of previously diagnosed chronic diseases that have shown a significant relation with death or functional outcomes in TBI population .[13-16] Given that there is no consensus on the most appropriate method to construct comorbidity in TBI or whether one type is preferred to another, this systematic review will set restrictions only towards comorbid disorders being diagnosed, excluding self-report. To account for inconsistencies between studies that will, at least partially, drive our results, close attention will be paid to definitions of comorbidity and assessment tools in each individual study when

For peer review only

analyzing and reporting results. In addition, the limiter zero-time (baseline assessment) will be set at six-months. This historical time limiter will be set to allow us to, indirectly, distinguish disorders that are chronic in nature (according to the World Health Organization, chronic disorders are those that require care beyond six months, such as diabetes, cardiovascular disorders, etc.), from those that may co-occur with TBI (neck injury, fractures, etc.) and those that develop as a result of a TBI or associated impairments, both physical and psychological, such as anxiety and/or mood disorders, infection disorders, etc. All attempts will be made to present results of acute comorbidity and chronic comorbidity associations with studied outcomes separately.

Predictors and outcomes

Predictors will be collated into three domains: sociodemographic characteristics, TBI-related characteristics, and comorbidity. All comorbidity-related variables (comorbidity index severity or presence of comorbidity) will be treated as primary predictors; hypothesized sociodemographic (age and sex) and TBI-related variables (injury severity, mechanism, and time since injury), if reported, will be considered as secondary predictors of our outcome(s) of interest. We will report on only those predictors that have been shown to be statistically significantly associated with our outcome(s) in at least one study and with reported quantitative data (i.e., event rates, risk ratios [RRs], odds ratios [ORs], or hazard ratios [HRs]) to measure the association between predictors and outcomes.

The following *outcomes* (either objectively documented or self-reported) will be considered: cognitive and physical status as assessed by the Glasgow Outcome Scale (GOS) with or without extended scores, Disability Rating Scale (DRS), Functional Independence Measure (FIM), the Functional Status Examination (FSE) or any other standardized functional measurement and

For peer review only - http://bmjopen.bmj.com/site/about/guidelines.xhtml

mortality. In order for the previous objective (i.e., functional status) to be considered, the study has to define at least two time points' scores of functioning and/or has to provide details on when (at which time point since injury) a loss/gain/plateau of function has been defined as a decline/improvement/stability. Should the definition of functional status vary amongst studies, the reported minimum change, expressed in percentage change, will be abstracted. For example, if the functional measure has a score in a range from zero to 10, and the minimum change reported by researchers for the sample was one, we will assign a 10% 'change' on this group's functional capabilities.

Inclusion and exclusion criteria

We will include studies that (1) primarily study comorbidity as it relates to our outcome(s) of interest; and (2) targeted adult patients (the mean age minus standard deviation is ≥18 years of age) with a diagnosis of TBI on the basis of the accepted definitions (not self-report), and followed them for any period of time. Studies of brain injury of only traumatic origin will be considered. Studies will neither be excluded based on the setting in which the research took place (acute care, rehabilitation setting, community, etc.) nor means of diagnosis of comorbidity. However, the following studies will be excluded: (1) more than 50% of participants had pre-existing TBIs or severe comorbidity (i.e., neurological or psychiatric diseases) at the baseline assessment, and if the subgroup with incident comorbidity and the patient outcome data could not be extracted independent of pre-existing cases (i.e., present before TBI); and (2) study designs/formats in letters to editors, reviews without data, case reports, or public reports, conference abstracts articles with no primary data, studies that focus on therapeutic interventions, and theses.

For peer review only - http://bmjopen.bmj.com/site/about/guidelines.xhtml

Zero-time (baseline assessment)

The nature of our objectives related to development of adverse outcomes in the TBI population (i.e., prognostic factors), raises the issue of zero-time bias. In prognostic studies, testing should start at a defined point, called zero time. Designated zero times (i.e. baseline or first assessment) vary between studies, [28] where the majority of research from acute care/emergency studies performed baseline assessment within the first month after injury and majority of rehabilitation or community studies of prognosis performed baseline assessment prior to or at six-months post injury mark.[29] The limiter to zero-time has been set at six-month.

Study selection

Two independent researchers (CX, SH) will assess study titles and abstracts. If the title or abstract suggest that the study might meet the inclusion criteria, both reviewers will assess the full article. Differences of opinion will be resolved by group discussion (CX, SH, and TM), with the goal to reach consensus in each case. Studies failing to meet the inclusion criteria will be excluded and the reason will be reported.

Data extraction and quality assessment

Study quality will be assessed independently by two researchers (TM, CX) using guidelines for assessing prognostic studies.[30] First, two researchers will independently assess the items related to potential sources of bias, namely: (i) study participation and attrition; (ii) prognostic factor and outcome measurements; (iii) confounding measurement and account; and (iv) analyses. Then, the same two reviewers will judge the presence of potential biases as “Yes”, “Partly”, “No”, or “Unsure”. Following these steps, the Scottish Intercollegiate Guidelines Network (SIGN) [31] methodology will be implemented where “++” will be assigned to each study when all or most of the quality criteria were fulfilled (allowing one “Partly” while appraising all potential sources

For peer review only - http://bmjopen.bmj.com/site/about/guidelines.xhtml

of bias); "+" when some of the criteria were fulfilled; and "–" when few or no criteria fulfilled (at least one "Yes"). In our review, we will refer to group "++" as 'high quality studies' and group "+" as 'moderate quality studies'. We will abstract data on the relationships between our outcomes of interest and primary (i.e., comorbidity) and secondary (i.e., socio-demographic, TBI-related) predictors only from studies with sufficient quality (i.e., 'high' and 'moderate' quality studies).[31]

Dealing with missing data

Primary authors will be contacted in cases of missing data. The proportion of missing data will be reported along with reasons where indicated. In the case of duplicate publications and companion papers of a primary study, we will attempt to yield maximum scientific information by abstraction of all available data. However, original publication (usually the earliest publication version) will take priority in data analysis.

Dealing with publication bias

The predisposition of journals to favour publication of positive reports over negative investigative findings and the reticence of authors to publish poor outcomes may lead to a high chance that results of studies included in this review may be affected by these publication biases. The most commonly used method to assess potential publication bias is the construction of a funnel plot, which is not an optimal methodology in highly heterogeneous studies that precludes expecting a symmetrical funnel shape.[32] To account for potential publication bias, we will apply an expert opinion methodology[33] to inform the study selection process. We will ask researchers and clinicians from our team with expertise in TBI about the probability of publication for small and large sample size studies that considered the effect (i.e., positive or negative) of any comorbidity in relationship to outcomes, and will report the average in their response. We will

For peer review only - http://bmjopen.bmj.com/site/about/guidelines.xhtml

then apply the selection model on the published studies and calculate an estimate from the published studies (without making any adjustments for publication bias). We will also report on the quality of the study and funding information. Such an approach is not confounded by heterogeneity as in the case of the funnel-plot approach.

Data synthesis and analysis

For the unadjusted analysis (Step 1), we will extract data from all studies that reported the effect of comorbidity on mortality and functional status and report number of events (death and functional decline/gain/plateau) relative to the total number of participants in the group with comorbid disorder(s) and control groups. The timeframe of follow- up assessments will be categorized into (i) short term, i.e., up to three months post injury mark; (ii) intermediate, i.e., 3-12 months (i.e., up to one year inclusive), and (iii) long-term, i.e., >1 year post-injury. These points were arbitrarily set. We reserve the right to adjust follow-up time frames based on time stratification applied in the included studies.

This stage will be followed by adjusted analysis (Step 2), where we will extract and analyze quantitative data (i.e., ORs, RRs, and HRs) that will be adjusted for hypothesized key confounders (age, sex, TBI mechanism and severity, and/or baseline functional status) reflecting the association between comorbidity and our outcomes of interest. Some of these variables, such as age, sex, and TBI severity are considered as key confounders, and will be included in our list of required adjusted variables for a study to be included in our primary analysis.[30] If the key variables are not included in the final adjusted model, control for confounding variables will be determined to be inadequate, which will be reflected in a risk of bias assessment table.[30] Where possible, we will perform a meta-analytic analysis: pooled-effect outcomes for each group of

For peer review only

comorbid disorders will be calculated using inverse variance methods with random-effects models[31] and expressed as ORs and 95% CIs. Heterogeneity will be assessed using the I^2 statistic. P values of 0.05 or less will be considered as statistically significant. In the case of statistical or clinical diversity in definitions of comorbidity and/or TBI, population of interest, and the statistical methodology used to quantify association in the studies, meta-analysis will not be performed and we will use a best-evidence synthesis approach, synthesizing findings from studies with sufficient quality through tabulation and qualitative description[34].

For peer review only

Discussion

Ethics and dissemination

This systematic review aims to investigate the relationship between comorbidity and functional outcomes and death within short, moderate and long-term time frames after TBI, as well as determining the prognostic value of comorbidity, sociodemographic (i.e., age, sex), and injury-related characteristics on these outcomes. The strength of this systematic review and research program is in its methodology, making it possible to identify associations longitudinally, thus improving the quality of inductive inferences regarding the natural progression of associative values of hypothesized predictors on outcomes in patients with TBI of various severities. Furthermore, our protocol was registered in PROSPERO and was designed in keeping with best-practice methods where the multilevel risk of bias assessment[26] will allow us to detect the main flaws in the individual studies' design and inform on the future research of comorbidities in TBI. Moreover, the clinical criteria for the diagnoses of TBI and comorbidity will be collected and reported, as it is expected that they have a significant impact on the study results. Finally, multi-

level knowledge translation activities throughout this research activity will be performed, ensuring that these results reach their intended knowledge users.

Limitations

The present study includes the following limitations: (1) the limiter of six months for zero-time may not be optimal as some comorbid disorder (demyelinating, degenerative, etc.) may take longer time to develop ; (2) the assumption of expected heterogeneity in the primary studies with respect to TBI-related characteristics (i.e., injury/localization of injury, time since injury) with severe TBI cases expect to be underrepresented; likewise severe comorbid disorders may precluded TBI patients with milder severities of injury to participate in research, limiting the precision of estimates of risk for severe TBI and comorbidity cases; (3) potentially unequal sex and age distribution in primary studies, given that historically TBI has been considered an injury of younger males and older females; (4) residual confounding due to TBI- or comorbidity-related treatment effect; (5) excepted complexity of assessing the risk of short-term functional change (i.e., fluctuation); and (6) additional limitations relate to the exclusion of grey literature, articles published in languages other than English, and limiting our searches to the past 20 years; this decision was based on the extensive number of studies identified within the databases searched, changes applied to clinical classifications and definitions of TBI, as well as limited empirical evidence about the potential impact of selective searching and inclusion of earlier works on the results of systematic reviews.[35]

Despite these limitations, this protocol is for a review, that is the first that comprehensively synthesizes evidence on prognostic value of comorbidity in TBI patients, aiming to enrich science and advance care provided to patients with comorbid disorders stemming from traumatic brain injuries.

For peer review only - http://bmjopen.bmj.com/site/about/guidelines.xhtml

Implications

The number of people surviving TBIs is increasing. While the neurological consequences of TBI are well described, evidence is emerging on associations between brain injury, comorbid disorders and adverse short and long-term outcomes post-injury.[36,37] The significant economic and human costs of TBIs merit the call for systematic efforts to understand all factors that contribute to adverse post-injury outcomes, including comorbidity;[38] all with the goal to allow better risk stratification to guide management of brain injury acutely and at the chronic stages post injury on a population level.

Funding sources

The study was funded by the National Institutes of Health (NIH) under Award 1R21 HD08106-01 (PI: Dr. Angela Colantonio). AC is supported by the Canadian Institutes for Health Research Grant–Institute for Gender and Health (#CGW-126580). TM is supported by the postdoctoral research grant Alzheimer's Association (AARF-16-442937). The funders had no role in study design, data collection, decision to publish, or preparation of the manuscript.

Competing interests

The authors have no conflict of interest to declare pertaining to this review.

Authors' contributions

Protocol concept and design: Tatyana Mollayeva, Angela Colantonio. Registry PROSPERO: Chen Xiong. Acquisition of data: Tatyana Mollayeva. Administrative, technical, and material support: Chen Xiong, Sara Hanafy. Statistical analysis approach: Vincy Chan, Zheng Jing Hu, Mitchell Sutton, Michael Escobar. Drafting of the manuscript: Tatyana Mollayeva. Critical revision of the manuscript for important intellectual content: All authors.

Human and Animal Rights and Informed Consent

For peer review only

This article does not contain any studies with human or animal subjects performed by any of the authors.

Acknowledgments

We gratefully acknowledge the involvement of Ms. Jessica Babineau, Information Specialist at the Toronto Rehabilitation Institute- University Health Network for her comprehensive literature search.

Additional material

File name: Supplementary TableS1.Search details

File format: Table S1_10072017.pdf

Title of data: Search strategies.

Description of data: This file provides the list of search terms used to search Medline, Central, Embase, Scopus, PsycINFO, and supplemental PubMed.

For peer review only

References

1. Brain Injury Association of America. About Brain Injury. Retrieved June12, 2017, from http://www.biausa.org/about-brain-injury.htm.
2. World Health Organization. 2006. Projections of Mortality and Burden of Disease, 2002−2030. Deaths by Income Group. [Computer file] Retrieved June 12, 2017, from <www.who.int/healthinfo/global_burden_disease/projections2002/en/>.
3. Coronado VG, McGuire LC, Sarmiento K, Bell J, Lionbarger MR, Jones CD, Geller AI, Khoury N, Xu L. Trends in Traumatic Brain Injury in the U.S. and the public health response: 1995-2009. *J Safety Res.* 2012;43(4):299-307.
4. Public Health Agency of Canada. 2014. Mapping Connections: An Understanding of Neurological Conditions in Canada. Ottawa, ON: Public Health Agency of Canada. Retrieved June 12, 2017, from <www.phac-aspc.gc.ca/publicat/cd-mc/mc-ec/assets/pdf/mc-ec-eng.pdf>.
5. Colantonio A, Saverino C, Zagorski B, Swaine B, Lewko J, Jaglal S et al. Hospitalizations and Emergency Department Visits for TBI in Ontario. *Can J Neuroll Sci*. 2010; 37(6): 783–90.
6. Feinstein AR. The pre-therapeutic classification of co-morbidity in chronic disease. *J Chron Disease.* 1970; 23: 455–68.
7. Chan V, Mollayeva T, Ottenbacher KJ, Colantonio A. Clinical profile and comorbidity of traumatic brain injury among younger and older men and women: a brief research notes. *BMC Res Notes*. 2017;10(1):371.

For peer review only

8. Brooks JC, Shavelle RM, Strauss DJ, Hammond FM, Harrison-Felix CL. Long-Term Survival After Traumatic Brain Injury Part II: Life Expectancy. *Arch Phys Med Rehabil.* 2015; 96(6):1000-5.

9. Fu TS, Jing R, McFaull SR, Cusimano MD. Recent trends in hospitalization and in-hospital mortality associated with traumatic brain injury in Canada: A nationwide, population-based study. *J Trauma Acute Care Surg.* 2015; 79(3 Suppl 1):449-55.

10. Popescu C, Anghelescu A, Daia C, Onose G. Actual data on epidemiological evolution and prevention endeavours regarding traumatic brain injury. *J Med Life.* 2015;8(3):272-7.

11. Valderas JM, Starfiels B, Sibbald B, Salisbury C, Roland M. Defining comorbidity: implications for understanding health and health services. *Ann Fam Med*. 2009; 7:357-63.

12. Gardizi E, Hanks RA, Millis SR, Figueroa MJ. Comorbidity and insurance as predictors of disability following traumatic brain injury. *Arch Phys Med Rehabil.* 2014; 95:2396–401.

13. Coronado VG, Thomas KE, Sattin RW, Johnson RL. The CDC traumatic brain injury surveillance system: Characteristics of persons aged 65 years and older hospitalized with a TBI. *J Head Trauma Rehabil.* 2005; 20(3): 215–28.

14. Colantonio A, Gerber G, Bayley M, Deber R, Yin J, Kim H. Differential profiles for patients with traumatic and non-traumatic brain injury. *J Rehabil Med.* 2011; 43(4): 311–15

15. Thompson HJ, Dikmen S, Temkin N. Prevalence of comorbidity and its association with traumatic brain injury and outcomes in older adults. *Res Gerontol Nurs.* 2012; 5(1): 17–24

For peer review only - http://bmjopen.bmj.com/site/about/guidelines.xhtml

16. Gardizi E, Hanks RA, Millis SR, Figueroa MJ. Comorbidity and insurance as predictors of disability following traumatic brain injury. *Arch Phys Med Rehabil.* 2014; 95(12): 2396–01.

17. Kumar RG, Juengst SB, Wang Z, Dams-O'Connor K, Dikmen SS, O'Neil-Pirozzi TM, Dahdah MN, Hammond FM, Felix ER, Arenth PM, Wagner AK. Epidemiology of Comorbid Conditions Among Adults 50 Years and Older With Traumatic Brain Injury. *J Head Trauma Rehabil.* 2017 [Epub ahead of print] doi: 10.1097/HTR.0000000000000273.

18. Fu TS, Jing R, McFaull SR, Cusimano MD. Recent trends in hospitalization and in-hospital mortality associated with traumatic brain injury in Canada: A nationwide, population-based study. *J Trauma Acute Care Surg.* 2015; 79(3):449-54.

19. Ventura T, Harrison-Felix C, Carlson N, Diguiseppi C, Gabella B, Brown A, Devivo M, Whiteneck G. Mortality after discharge from acute care hospitalization with traumatic brain injury: a population-based study. *Arch Phys Med Rehabil.* 2010;91(1):20-9.

20. Hodgkinson A, Veerabangsa A, Drane D, McCluskey A. Service utilization following traumatic brain injury. *J Head Trauma Rehabil.* 2000;15(6):1208-26.

21. Perrin PB, Niemeier JP, Mougeot JL, Vannoy CH, Hirsch MA, Watts JA, Rossman W, Grafton LM, Guerrier TD, Pershad R, Kingsbury CA, Bartel SW, Whitney MP. Measures of injury severity and prediction of acute traumatic brain injury outcomes. *J Head Trauma Rehabil.* 2015; 30(2):136-42.

22. Krasny-Pacini A, Chevignard M, Lancien S, Escolano S, Laurent-Vannier A, De Agostini M, Meyer P. Executive function after severe childhood traumatic brain injury - Age-at-

For peer review only - http://bmjopen.bmj.com/site/about/guidelines.xhtml

injury vulnerability periods: The TGE prospective longitudinal study. *Ann Phys Rehabil Med.* 2017;60(2):74-82.

23. Klauber MR, Marshall LF, Barrett-Connor E, et al: Prospective study of patients hospitalized with head injury in San Diego County, 1978. *Neurosurgery.* 1981; 9:236–41.
24. Slewa-Younan S, van den Berg S, Baguley IJ, Nott M, Cameron ID. Towards an understanding of sex differences in functional outcome following moderate to severe traumatic brain injury: a systematic review. *J Neurol Neurosurg Psychiatry*. 2008;79(11):1197-201.
25. Moher D, Liberati A, Tetzlaff J, Altman DG. Preferred reporting items for systematic reviews and meta-analyses for PRISMA statement. *PLoS Medicine.* 2009; 6(7).
26. PROSPERO- International Prospective Register of Systematic Reviews. [http://www.crd.york.ac.uk/prospero/].
27. Valderas JM, Starfiels B, Sibbald B, Salisbury C, Roland M. Defining comorbidity: implications for understanding health and health services. *Ann Fam Med.* 2009; 7:357-363.
28. Canadian Coding Standards for Version 2015 ICD-10-CA and CCI, assessed August 25, 2017 at: https://secure.cihi.ca/free_products/Coding%20standard_EN_web.pdf
29. Mollayeva T, Kendzerska T, Mollayeva S, Shapiro CM, Colantonio A, Cassidy JD. A systematic review of fatigue in patients with traumatic brain injury: the course, predictors and consequences. *Neurosci Biobehav Rev.* 2014;47: 684-716.
30. Hayden JA, Cote P, Bombardier C. Evaluation of the quality of prognosis studies in systematic reviews. *Ann Intern Med.* 2006; 144: 427-37.

For peer review only

31. Scottish Intercollegiate Guidelines Network: Published Guidelines. Retrieved 03 09 2017 from http://www.sign.ac.uk.guidelines/

32. Terrin N, Schmid CH, Lau J. In an empirical evaluation of the funnel plot, researchers could not visually identify publication bias. *J Clin Epidemiol.* 2005; 58(9):894-901.

33. Mavridis D, Salanti G. Exploring and accounting for publication bias in mental health: a brief overview of methods. *Evid Based Ment Health*. 2014; 17(1):11-5.

34. Slavin RE. (1995) Best evidence synthesis: an intelligent alternative to meta-analysis. J Clin Epidemiol, 48: 9–18.

35. Hartling L, Featherstone R, Nuspl M, Shave K, Dryden DM, Vandermeer B. Grey literature in systematic reviews: a cross-sectional study of the contribution of non-English reports, unpublished studies and dissertations to the results of meta-analyses in child-relevant reviews. Reference: *BMC Med Res Methodol.* 2017;17(1):64.

36. Shukla D, Devi BI, Agrawal A. Outcome measures for traumatic brain injury. *Clin Neurol Neurosurg*. 2011; 113(6):435-41.

37. Nichol AD, Higgins AM, Gabbe BJ, Murray LJ, Cooper DJ, Cameron PA. Measuring functional and quality of life outcomes following major head injury: common scales and checklists. *Injury.* 2011;42(3):281-7.

38. Charlson ME, Pompei P, Ales KL, MacKenzie CR. A new method of classifying prognostic comorbidity in longitudinal studies: development and validation. *J Chronic Dis.* 1987;40(5):373-83.

For peer review only

Supplementary Table S1. Search details.
Searches conducted in Medline (including Medline in Process and other non-indexed citations, ePubs and Medline Daily), Embase, Cochrane Central Register of Controlled Trials, and PsycINFO. Searches were limited from 1997 to May 2017. Searches were limited to English language papers, and to an adult, human population when possible. Searches were conducted by an Information Specialist (JB).

PRE-DUPLICATE REMOVAL
TOTAL Results: 9414
Medline: 2860
Central: 178
Embase: 5468
PsycINFO: 908

POST-DUPLICATE REMOVAL
TOTAL Results: 7443
Duplicates removed: 1971
Medline: 2748
Central: 116
Embase: 4088
PsycINFO: 491

Database: Ovid MEDLINE(R) Epub Ahead of Print, In-Process & Other Non-Indexed Citations, Ovid MEDLINE(R) Daily and Ovid MEDLINE(R) <1946 to Present>
Search Strategy:
----
1 exp brain injuries/ (59169)
2 Craniocerebral Trauma/ (21199)
3 exp Head Injuries, Closed/ (9232)
4 exp Skull Fractures/ (20700)
5 mTBI*2.tw,kw. (1835)
6 tbi*2.tw,kw. (20636)
7 concuss*.tw,kw. (6496)
8 ((head* or cerebr* or crani* or skull* or intracran*) adj2 (injur* or trauma* or damag* or wound* or swell* or oedema* or edema* or fracture* or contusion* or pressur*)).tw,kw. (75791)
9 ((brain* or cerebr* or intracerebr* or crani* or intracran* or head* or subdural* or epidural* or extradural*) adj (haematoma* or hematoma* or hemorrhag* or haemorrhag* or bleed*)).tw,kw. (42638)
10 or/1-9 (185742)
11 exp Comorbidity/ (88810)
12 exp Risk Adjustment/ (2770)
13 (comorbid* or co morbid* or multimorbid* or multi morbid*).tw,kw. (131913)
14 (polypatholog* or poly-patholog*).tw,kw. (151)
15 ((clinical* or medical*) adj3 complex*).tw,kw. (11314)

For peer review only

16 ((coexist* or co exist* or cooccur* or co-occur* or multipl*) adj3 (illness* or disease* or disorder* or condition* or complication* or diagnos* or risk*)).tw,kw. (69114)
17 (multidisease? or multi-disease? or (multiple adj (ill* or disease? or condition? or syndrom* or disorder?))).tw,kw. (3721)
18 ((several* or various or (two adj2 more) or concomitant or conjoined or concurrent) adj3 (morbid* or ill* or disease* or sick* or condition*)).tw,kw. (129147)
19 "comorbidity-polypharmacy score".tw,kw. (11)
20 ('charlson comorbidity index' or 'CCI' or 'CMI' or elixhauser or 'BOD index' or 'cumulative index rating scale' or 'CIRS' or 'Coroni-Huntley index' or 'DUSOI index' or 'Hallstrom index' or 'Hurwitz index' or 'Incalzi index', 'Kaplan index', 'Liu index', 'Shwartz index' or 'comorbidity-polypharmacy score').tw,kw. (10394)
21 or/11-20 (394772)
22 10 and 21 (5122)
23 exp Mortality/ (335478)
24 exp morbidity/ (467773)
25 (morbidit* or mortalit*).tw,kw. (760891)
26 function*.mp. (3282516)
27 or/23-26 (4495784)
28 exp cohort studies/ (1680971)
29 exp prognosis/ (1379668)
30 exp survival analysis/ (241795)
31 exp models, statistical/ (344993)
32 prognos*.tw,kw. (492733)
33 predict*.tw,kw. (1261259)
34 course*.tw,kw. (546731)
35 diagnosed.tw,kw. (461120)
36 cohort*.tw,kw. (423751)
37 death.tw,kw. (584394)
38 exp treatment outcome/ (841041)
39 "early termination of clinical trials"/ (544)
40 treatment failure/ (31446)
41 incidence/ (222806)
42 or/28-41 (5055925)
43 27 or 42 (8021818)
44 22 and 43 (3756)
45 44 not (exp animals/ not exp humans/) (3301)
46 45 not (exp children/ not exp adults/) (3118)
47 limit 46 to english language (2860)
48 remove duplicates from 47 (2750)

****
Database: Embase <1974 to 2017 May 10>
Search Strategy:
----
1 exp brain injury/ (148571)

For peer review only

2 head injury/ (44023)
3 mTBI*2.tw. (2763)
4 tbi*2.tw. (32342)
5 concuss*.tw. (7543)
6 ((head* or cerebr* or crani* or skull* or intracran*) adj2 (injur* or trauma* or damag* or wound* or swell* or oedema* or edema* or fracture* or contusion* or pressur*)).tw,kw. (96428)
7 ((brain* or cerebr* or intracerebr* or crani* or intracran* or head* or subdural* or epidural* or extradural*) adj (haematoma* or hematoma* or hemorrhag* or haemorrhag* or bleed*)).tw,kw. (59251)
8 or/1-7 (291625)
9 comorbidity/ (177225)
10 risk assessment/ (409214)
11 exp comorbidity assessment/ (9540)
12 (comorbid* or co morbid* or multimorbid* or multi morbid*).tw,kw. (217271)
13 (polypatholog* or poly-patholog*).tw,kw. (290)
14 ((clinical* or medical*) adj3 complex*).tw,kw. (15500)
15 ((coexist* or co exist* or cooccur* or co-occur* or multipl*) adj3 (illness* or disease* or disorder* or condition* or complication* or diagnos* or risk*)).tw,kw. (94039)
16 (multidisease? or multi-disease? or (multiple adj (ill* or disease? or condition? or syndrom* or disorder?))).tw,kw. (4710)
17 ((several* or various or (two adj2 more) or concomitant or conjoined or concurrent) adj3 (morbid* or ill* or disease* or sick* or condition*)).tw,kw. (163497)
18 "comorbidity-polypharmacy score".tw,kw. (15)
19 ('charlson comorbidity index' or 'CCI' or 'CMI' or elixhauser or 'BOD index' or 'cumulative index rating scale' or 'CIRS' or 'Coroni-Huntley index' or 'DUSOI index' or 'Hallstrom index' or 'Hurwitz index' or 'Incalzi index', 'Kaplan index', 'Liu index', 'Shwartz index' or 'comorbidity-polypharmacy score').tw,kw. (19018)
20 or/9-19 (943533)
21 8 and 20 (15388)
22 exp mortality/ (858109)
23 exp morbidity/ (299827)
24 (morbidit* or mortalit*).tw,kw. (1038507)
25 function*.mp. (4279406)
26 or/22-25 (5472712)
27 cohort analysis/ (287239)
28 prognosis/ (506894)
29 survival analysis/ (3712)
30 exp survival/ (845725)
31 statistical model/ (136685)
32 exp "prediction and forecasting"/ (1010760)
33 prognos*.tw,kw. (700803)
34 predict*.tw,kw. (1580756)
35 course*.tw,kw. (702204)
36 diagnosed.tw,kw. (688998)

For peer review only

For peer review only - http://bmjopen.bmj.com/site/about/guidelines.xhtml

37 cohort*.tw,kw. (649460)
38 death.tw,kw. (778611)
39 exp treatment outcome/ (1236966)
40 "early termination of clinical trial"/ (204)
41 exp treatment failure/ (108708)
42 exp incidence/ (330423)
43 or/27-42 (5901375)
44 26 or 43 (9681606)
45 21 and 44 (10931)
46 45 not ((exp animals/ or exp animal experimentation/ or nonhuman/) not exp human/) (9877)
47 46 not ((exp embryo/ or exp fetus/ or exp juvenile/) not exp adult/) (8968)
48 limit 47 to english language (8463)
49 limit 48 to embase (5871)
50 limit 49 to (conference abstract or conference paper or "conference review") (179)
51 49 not 50 (5692)
52 limit 51 to yr="1997 -Current" (5468)

****

Database: EBM Reviews - Cochrane Central Register of Controlled Trials <April 2017>
Search Strategy:
----
1 exp brain injuries/ (1135)
2 Craniocerebral Trauma/ (247)
3 exp Head Injuries, Closed/ (167)
4 exp Skull Fractures/ (192)
5 mTBI*2.tw,kw. (122)
6 tbi*2.tw,kw. (1306)
7 concuss*.tw,kw. (221)
8 ((head* or cerebr* or crani* or skull* or intracran*) adj2 (injur* or trauma* or damag* or wound* or swell* or oedema* or edema* or fracture* or contusion* or pressur*)).tw,kw. (3278)
9 ((brain* or cerebr* or intracerebr* or crani* or intracran* or head* or subdural* or epidural* or extradural*) adj (haematoma* or hematoma* or hemorrhag* or haemorrhag* or bleed*)).tw,kw. (3605)
10 or/1-9 (8539)
11 exp Comorbidity/ (3012)
12 exp Risk Adjustment/ (20)
13 (comorbid* or co morbid* or multimorbid* or multi morbid*).tw,kw. (9750)
14 (polypatholog* or poly-patholog*).tw,kw. (4)
15 ((clinical* or medical*) adj3 complex*).tw,kw. (711)
16 ((coexist* or co exist* or cooccur* or co-occur* or multipl*) adj3 (illness* or disease* or disorder* or condition* or complication* or diagnos* or risk*)).tw,kw. (4140)
17 (multidisease? or multi-disease? or (multiple adj (ill* or disease? or condition? or syndrom* or disorder?))).tw,kw. (107)

For peer review only - http://bmjopen.bmj.com/site/about/guidelines.xhtml

18 ((several* or various or (two adj2 more) or concomitant or conjoined or concurrent) adj3 (morbid* or ill* or disease* or sick* or condition*)).tw,kw. (1145)
19 "comorbidity-polypharmacy score".tw,kw. (0)
20 ('charlson comorbidity index' or 'CCI' or 'CMI' or elixhauser or 'BOD index' or 'cumulative index rating scale' or 'CIRS' or 'Coroni-Huntley index' or 'DUSOI index' or 'Hallstrom index' or 'Hurwitz index' or 'Incalzi index', 'Kaplan index', 'Liu index', 'Shwartz index' or 'comorbidity-polypharmacy score').tw,kw. (748)
21 or/11-20 (17592)
22 10 and 21 (214)
23 22 not (exp animals/ not exp humans/) (214)
24 23 not (exp Child/ not exp Adult/) (213)
25 limit 24 to english language (185)
26 limit 25 to yr="1997 -Current" (178)

****

Database: PsycINFO <1806 to May Week 1 2017>
Search Strategy:
----
1 exp traumatic brain injury/ (15677)
2 exp head injuries/ (5584)
3 mTBI*2.tw. (1312)
4 tbi*2.tw. (8651)
5 concuss*.tw. (2360)
6 ((head* or cerebr* or crani* or skull* or intracran*) adj2 (injur* or trauma* or damag* or wound* or swell* or oedema* or edema* or fracture* or contusion* or pressur*)).tw. (11318)
7 ((brain* or cerebr* or intracerebr* or crani* or intracran* or head* or subdural* or epidural* or extradural*) adj (haematoma* or hematoma* or hemorrhag* or haemorrhag* or bleed*)).tw. (3029)
8 or/1-7 (28382)
9 comorbidity/ (27127)
10 risk assessment/ (11895)
11 (comorbid* or co morbid* or multimorbid* or multi morbid*).tw. (49497)
12 (polypatholog* or poly-patholog*).tw. (23)
13 ((clinical* or medical*) adj3 complex*).tw. (2577)
14 ((coexist* or co exist* or cooccur* or co-occur* or multipl*) adj3 (illness* or disease* or disorder* or condition* or complication* or diagnos* or risk*)).tw. (16826)
15 (multidisease? or multi-disease? or (multiple adj (ill* or disease? or condition? or syndrom* or disorder?))).tw. (666)
16 ((several* or various or (two adj2 more) or concomitant or conjoined or concurrent) adj3 (morbid* or ill* or disease* or sick* or condition*)).tw. (14708)
17 "comorbidity-polypharmacy score".tw. (2)
18 ('charlson comorbidity index' or 'CCI' or 'CMI' or elixhauser or 'BOD index' or 'cumulative index rating scale' or 'CIRS' or 'Coroni-Huntley index' or 'DUSOI index' or

For peer review only - http://bmjopen.bmj.com/site/about/guidelines.xhtml

'Hallstrom index' or 'Hurwitz index' or 'Incalzi index', 'Kaplan index', 'Liu index', 'Shwartz index' or 'comorbidity-polypharmacy score').tw,tm. (2701)
19 or/9-18 (97253)
20 8 and 19 (1354)
21 limit 20 to animal (317)
22 limit 20 to human (1042)
23 20 not (21 not 22) (1071)
24 limit 23 to ((childhood <birth to 12 years> or adolescence <13 to 17 years>) and (100 childhood <birth to age 12 yrs> or 120 neonatal <birth to age 1 mo> or 140 infancy <2 to 23 mo> or 160 preschool age <age 2 to 5 yrs> or 180 school age <age 6 to 12 yrs> or 200 adolescence <age 13 to 17 yrs>)) (132)
25 limit 23 to (adulthood <18+ years> and ("300 adulthood <age 18 yrs and older>" or 320 young adulthood <age 18 to 29 yrs> or 340 thirties <age 30 to 39 yrs> or 360 middle age <age 40 to 64 yrs> or "380 aged <age 65 yrs and older>" or "390 very old <age 85 yrs and older>")) (589)
26 23 not (24 not 25) (1023)
27 limit 26 to english language (977)
28 limit 27 to yr="1997 -Current" (908)

****

For peer review only

For peer review only - http://bmjopen.bmj.com/site/about/guidelines.xhtml

PRISMA-P (Preferred Reporting Items for Systematic review and Meta-Analysis Protocols) 2015 checklist: recommended items to address in a systematic review protocol*

Section and topic	Item No	Checklist item	Location; page(s) within the manuscript
**ADMINISTRATIVE INFORMATION**			
Title:			
Identification	1a	Identify the report as a protocol of a systematic review	Title, abstract, manuscript; pgs. 1-18
Update	1b	If the protocol is for an update of a previous systematic review, identify as such	NA; original review protocol
Registration	2	If registered, provide the name of the registry (such as PROSPERO) and registration number	PROSPERO: CRD42017070033
Authors:			
Contact	3a	Provide name, institutional affiliation, e-mail address of all protocol authors; provide physical mailing address of corresponding author	Title page: 1-2
Contributions	3b	Describe contributions of protocol authors and identify the guarantor of the review	Pgs.20
Amendments	4	If the protocol represents an amendment of a previously completed or published protocol, identify as such and list changes; otherwise, state plan for documenting important protocol amendments	N/A
Support:			
Sources	5a	Indicate sources of financial or other support for the review	Pg. 20
Sponsor	5b	Provide name for the review funder and/or sponsor	Pg. 20
Role of sponsor or funder	5c	Describe roles of funder(s), sponsor(s), and/or institution(s), if any, in developing the protocol	Pg. 20
**INTRODUCTION**			
Rationale	6	Describe the rationale for the review in the context of what is already known	Pgs. 8-9
Objectives	7	Provide an explicit statement of the question(s) the review will address with reference to participants, interventions, comparators, and outcomes (PICO)	Pg. 9
**METHODS**			

For peer review only

Eligibility criteria	8	Specify the study characteristics (such as PICO, study design, setting, time frame) and report characteristics (such as years considered, language, publication status) to be used as criteria for eligibility for the review	Pg. 11, pg. 14
Information sources	9	Describe all intended information sources (such as electronic databases, contact with study authors, trial registers or other grey literature sources) with planned dates of coverage	Pg. 10
Search strategy	10	Present draft of search strategy to be used for at least one electronic database, including planned limits, such that it could be repeated	Supplementary file Table S1
Study records:			
Data management	11a	Describe the mechanism(s) that will be used to manage records and data throughout the review	Pg. 14-15
Selection process	11b	State the process that will be used for selecting studies (such as two independent reviewers) through each phase of the review (that is, screening, eligibility and inclusion in meta-analysis)	Pg. 14
Data collection process	11c	Describe planned method of extracting data from reports (such as piloting forms, done independently, in duplicate), any processes for obtaining and confirming data from investigators	Pg. 14-16
Data items	12	List and define all variables for which data will be sought (such as PICO items, funding sources), any pre-planned data assumptions and simplifications	Pgs.14-17
Outcomes and prioritization	13	List and define all outcomes for which data will be sought, including prioritization of main and additional outcomes, with rationale	Pg. 13-14
Risk of bias in individual studies	14	Describe anticipated methods for assessing risk of bias of individual studies, including whether this will be done at the outcome or study level, or both; state how this information will be used in data synthesis	Pgs.15-16
Data synthesis	15a	Describe criteria under which study data will be quantitatively synthesised	Pg. 17
	15b	If data are appropriate for quantitative synthesis, describe planned summary measures, methods of handling data and methods of combining data from studies, including any planned exploration of consistency (such as I^2, Kendall's τ)	Pg. 17
	15c	Describe any proposed additional analyses (such as sensitivity or subgroup analyses, meta-regression)	Pg. 17
	15d	If quantitative synthesis is not appropriate, describe the type of summary planned	Pg.18

For peer review only

Meta-bias(es)	16	Specify any planned assessment of meta-bias(es) (such as publication bias across studies, selective reporting within studies)	Pg.16
Confidence in cumulative evidence	17	Describe how the strength of the body of evidence will be assessed (such as GRADE)	Pg. 15-16

*** It is strongly recommended that this checklist be read in conjunction with the PRISMA-P Explanation and Elaboration (cite when available) for important clarification on the items. Amendments to a review protocol should be tracked and dated. The copyright for PRISMA-P (including checklist) is held by the PRISMA-P Group and is distributed under a Creative Commons Attribution Licence 4.0.**

From: Shamseer L, Moher D, Clarke M, Ghersi D, Liberati A, Petticrew M, Shekelle P, Stewart L, PRISMA-P Group. Preferred reporting items for systematic review and meta-analysis protocols (PRISMA-P) 2015: elaboration and explanation. BMJ. 2015 Jan 2;349(jan02 1):g7647.

For peer review only
